# A modest protective association between pet ownership and cardiovascular diseases: A systematic review and meta-analysis

**DOI:** 10.1371/journal.pone.0216231

**Published:** 2019-05-03

**Authors:** Tzu-Lin Yeh, Wei-Te Lei, Shu-Jung Liu, Kuo-Liong Chien

**Affiliations:** 1 Department of Family Medicine, Hsinchu MacKay Memorial Hospital, Hsinchu City, Taiwan; 2 Institute of Epidemiology and Preventive Medicine, College of Public Health, National Taiwan University, Taipei City, Taiwan; 3 Department of Pediatrics, Hsinchu MacKay Memorial Hospital, Hsinchu, Taiwan; 4 Graduate Institute of Clinical Medical Sciences, College of Medicine, Chang Gung University, Taoyuan City, Taiwan; 5 Department of Medical Library, MacKay Memorial Hospital, Tamsui Branch, New Taipei City, Taiwan; 6 Department of Internal Medicine, National Taiwan University Hospital, Taipei, Taiwan; Karolinska Institutet, SWEDEN

## Abstract

**Purpose:**

Investigate the relationship between pet ownership and cardiovascular (CV) outcomes.

**Methods:**

We searched the PubMed, Ovid EMBASE, Cumulative Index to Nursing and Allied Health Literature, Cochrane Database of Systematic Reviews, and Cochrane Central Register of Controlled Trials databases up to August 2018. Eligible publications examining the association between pet ownership and all-cause and CV mortality (primary outcomes) and risks of cardiovascular disease (CVD), myocardial infarction (MI), and stroke (secondary outcomes) were included. We used the Newcastle–Ottawa Scale to assess the quality of the articles.

**Results:**

We included 12 studies, involving 488,986 participants (52.3% female, mean age 56.1 years), in our systematic review. The mean follow-up duration was 8.7 ± 6.3 years. Pet ownership had no association with adjusted all-cause mortality (odds ratio, OR = 1.01, 95% confidence interval, CI [0.94, 1.08], *I*^*2*^ = 76%), adjusted CV mortality (OR = 0.87, 95% CI [0.75, 1.00], *I*^*2*^ = 72%), or risk of cardiovascular disease (CVD) (OR = 0.87, 95% CI [0.72, 1.05], *I*^*2*^ = 73%), myocardial infarction (MI) (OR = 0.99, 95% CI [0.97, 1.01], *I*^*2*^ = 0%), or stroke (OR = 0.99, 95% CI [0.98, 1.01], *I*^*2*^ = 0%). However, subgroup analysis showed that pet ownership was associated with a lower adjusted CV mortality in the general population (OR = 0.93, 95% CI [0.86, 0.99], *I*^*2*^ = 27%) than in CVD patients. In patients with established CVD, pet ownership was associated with a lower adjusted CVD risk (OR = 0.71, 95% CI [0.60, 0.84], *I*^*2*^ = 0%).

**Conclusion:**

Pet ownership is not associated with adjusted all-cause or CV mortality, or risk of CVD, MI, or stroke, but it is associated with a lower adjusted CV mortality in the general population and a lower CVD risk in patients with established CVD.

## Introduction

Cardiovascular disease (CVD) is the global leading cause of death. CVD encompasses four major areas: (1) coronary heart disease (CHD), manifested by myocardial infarction (MI), angina pectoris, and heart failure; (2) cerebrovascular disease, manifested by stroke and transient ischemic attack; (3) peripheral artery disease; and (4) aortic atherosclerosis and aortic aneurysm. An estimated 17.7 million people died from CVDs in 2015, representing 31% of all global deaths[1]. CHD represents half of the total number of CVD events[2]. As the incidence of CVDs accelerates, the need for a more focused response is increasing.

Pet ownership, which means owning a pet and living together with the pet in a household, is popular worldwide. A total of 83% of Australians have had a pet in their lives[3]. Since the 1970s, the number of people with pet ownership in the United States (US) has more than tripled. About 85 million families, or 68% of US households, own a pet[4]. A study has shown that pet ownership and interactions with pets are associated with positive physical and mental health, demonstrating a correlation between human-animal interactions and improved physical and mental health[5]. Another study showed that pet ownership resulted in improvements in cardiovascular (CV) outcomes by providing social support and motivation for physical activity[6].

The American Heart Association released a scientific statement in 2015 that focused on pet ownership and established CV risk factors, including hypertension, hyperlipidemia, physical activity, obesity, autonomic function, CV reactivity, and, most importantly, survival with or without established CVD[7]. They concluded that pet ownership, particularly dog ownership, may have some causal role in reducing CVD risk (Level of Evidence: B). However, after this statement was published, large cohort studies addressing this topic have shown conflicting results. A large pooled analysis in 2018 of six population-based cohorts in England during 679,441 person-years follow-up showed no evidence for an association between living with a dog and all-cause or CV mortality[8]. Another large national Swedish cohort study published in 2017 revealed that dog ownership was significantly associated with a lower risk of death, CVD, and CV mortality[9]. This new evidence was contrary to that of a previous study in 2010, which showed that cat ownership was associated with increased cardiac morbidity and mortality one year following an admission for an acute coronary syndrome[10].

Our aim is to perform an updated systematic review and meta-analysis, incorporating all possible previous studies to verify the reported inconsistencies, and to evaluate the relationship between pet ownership and CVD and CV mortality. Our primary outcomes were CV mortality and all-cause mortality, while our secondary outcomes were CVD, including CHD, MI, and stroke risks.

## Methods

This systematic review and meta-analysis were conducted in accordance with the PRISMA-P guidelines[11] (S1 Table).

### Data sources and search strategy

We searched the following databases from study inception to August 2018: PubMed, Ovid EMBASE, the Cumulative Index to Nursing and Allied Health Literature(CINAHL), and the Cochrane database. We used the following keywords: (pets OR dogs OR cats OR animal) AND (ownership OR companion OR owning) AND (mortality OR fatality OR death rate OR cardiovascular diseases OR coronary disease OR myocardial ischemia OR heart attack). We did not limit the parameters of language, article type, year of publication, animal or human subjects, and age of participants to enable a comprehensive search. Tzu Lin Yeh and Shu Jung Liu conducted these searches independently, and disagreements were resolved through discussion with the third author, Wei Te Lei. The search strategies are shown in S1 Appendix.

### Study selection and methodological quality assessment

We included all eligible publications that followed our inclusion criteria: (1) the participants were with or without established disease, including the general healthy population and participants with established CVD; (2) pet ownership was compared with non-pet ownership; (3) any kind of pet ownership, including of dogs, cats, birds, or other, was investigated; (4) publications examining the association between pet ownership and all-cause and CV mortality and risks of CVD, MI, and stroke, were included, either as a primary or secondary outcome of the paper; (5) the articles on cohort, case-control, or randomized controlled trials contained data that could be extracted. We excluded articles that were (1) duplicate publications; (2) irrelevant to the topic; (3) non-specific in defining pet ownership, for example, only analyzing the duration of playing with animals or intermittent human-animal interactions such as animal-assisted therapies; (4) based on outcome measures that were other than mortality or CVD, such as physical activity, blood pressure, blood lipid profiles, heart rate variability, psychological, or anthropometric measures; (5) review articles or case reports.

**Fig 1:**
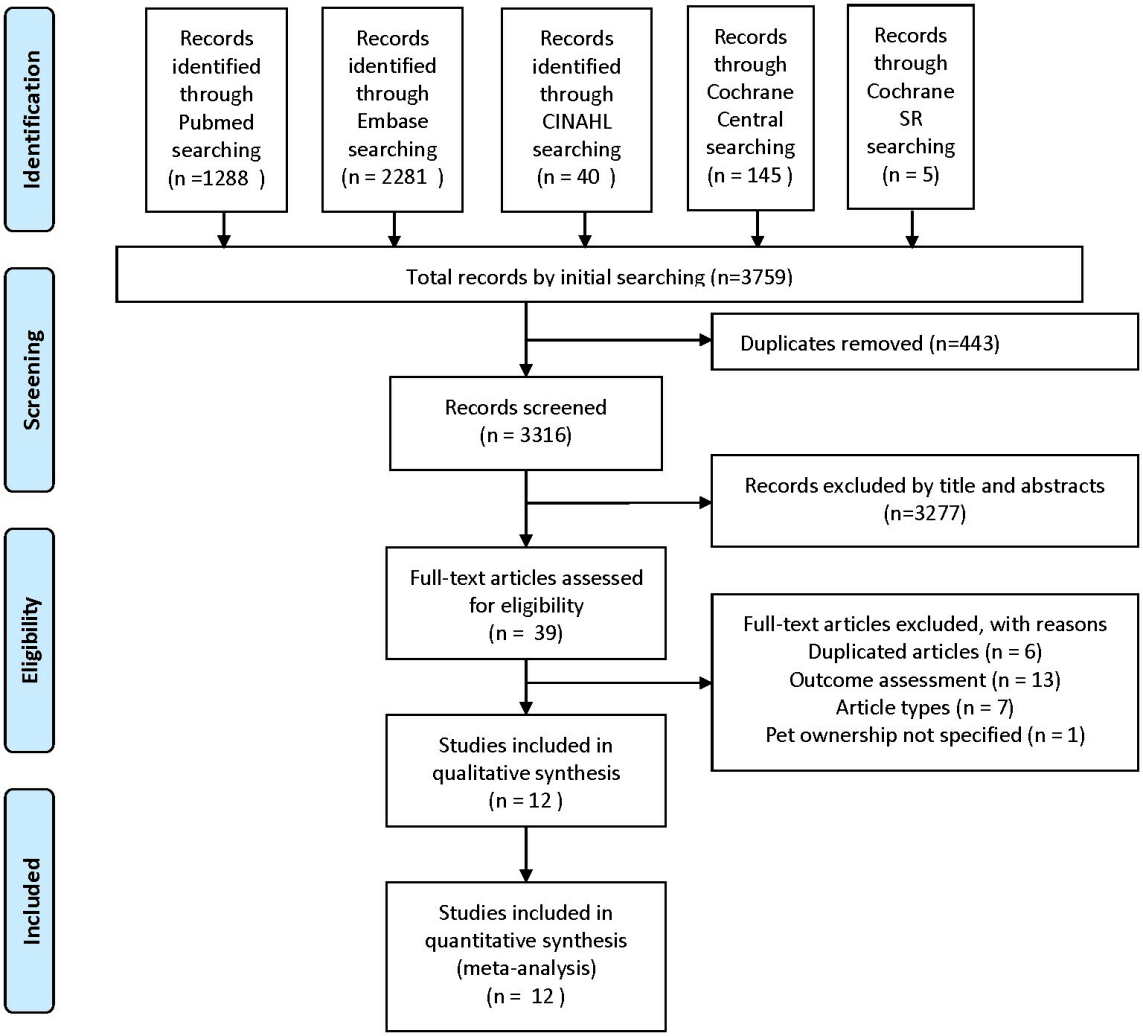
Flowchart of the study selection process. CINAHL, Cumulative Index to Nursing and Allied Health Literature; SR, systematic review.

Authors Tzu Lin Yeh and Wei Te Lei independently used the Newcastle-Ottawa Scale, a tool used for assessing the quality of non-randomized studies included in a systematic review. The scale includes the quality of selection (representativeness of the exposed cohort, selection of the non-exposed cohort, ascertainment of exposure, and demonstration that the outcome measure was not present at the start of study); comparability (comparability of cohorts on the basis of the design or analysis); and outcome (assessment of the outcome, if the follow-up period was long enough for outcomes to occur, and adequacy of the follow-up of cohorts)[12]. If the two authors had different opinions while assessing the studies, agreement was reached by consensus with the third author, Kuo Liong Chien. The study flow diagram is shown in Fig 1.

### Data extraction and analysis

Tzu Lin Yeh and Wei Te Lei independently extracted the following data from all included studies: last name of the first author, year of publication, characteristics of participants, type of pet ownership, outcome measurements, and the major findings (Table 1). During data extraction, we did not analyze the results of the crude rate of mortality and risk of disease. For observational studies, different adjustments were performed across these studies. If the original paper had several adjusted rates according to different models, we extracted data from the model adjusted for the most variables to make sure that the pet owners and non-pet owners had the most balanced socioeconomic conditions.

**Table 1:** Characteristics of included studies.

Reference	Country	Study Design	Pet Type	n (pet owner: non-pet owner)	Percentage of Women (pet owner: non-pet owner)	Mean Age (SD) (pet owner: non-pet owner)	Outcome Measure	Follow-up Duration Mean (SD)	Findings
**Ding 2018** [8]	England	A pooled analysis of six population-based cohorts	Dog	17,071: 42,281	54.8: 54.1	44.5 (16.2): 47.3 (18.5)^a^	All-cause and CV mortality	11.5(3.8) years	Dog ownership and all-cause mortality, HR = 1.03, 95% CI = 0.98–1.09 Dog ownership and CV mortality, HR = 1.07, 95% CI = 0.96–1.18
**Xie 2017** [16]	China	Cross-sectional study of patients hospitalized for coronary arteriography	Dog, cat	110: 451	23.6: 35.3	62.1 (8.9): 62.0(10.6)	CVD (CHD)	N/A	Reduced CHD risk among dog owners (OR = 0.42, 95% CI = 0.24–0.73^a^)
**Torske 2017** [17]	Norway	HUNT2, 1995±1997	Dog	10, 668: 42,750	52.8:54.5	46.7(14.1): 51.2(17.7)	All-cause mortality or physical activity	18.5 years (median)	Mortality is not significantly decreased in dog owners (HR = 1.00, 95% CI = 0.91–1.09)
**Mubanga 2017** [9]	Swedish	National cohort	Dog	448,298: 2,983,855	51.1: 52.4	51.7(8.2): 57.9(11.1)	All-cause, CV mortality and CVD	up to 12 years	Significantly lower all-cause mortality in dog owners (HR = 0.67, 95% CI = 0.65–0.69 ^a^), CV mortality (HR = 0.64, 95% CI = 0.59–0.70 ^a^), and CVD (HR = 0.92, 95% C = 0.89–0.94^a^)
		Swedish Twin Registry		2, 909: 31,293	60.5: 54.7	53.3 (7.7): 57.8 (9.8)		up to 14 years (total)	No difference in risk of CVD (HR = 1.09, 95% CI = 0.93–1.29) or all-cause mortality (HR = 0.87, 95% CI = 0.71–1.07) between dog owners and non-dog owners.
**Chowdhury 2017** [18]	Australian	ANBP2	Dog, cat, bird, fish, horse, other	1456: 549	48.6: 46.3	70.7(4.6): 72.6(5.0)	All-cause and CV mortality	Median 10.9 (IQR 10.2–11.4) years	All-cause mortality of pet owners, HR = 0.84, 95% CI = 0.71–1.00 ^a^ CV mortality of pet owners, HR = 0.74, 95% CI = 0.57–0.96
**Ogechi 2016** [19]	US	NHANES III, 1988–1994	Dog, cat	1215: 2749^b^	52.1: 52.7	63.1 ^a^	CV mortality, CVD (stroke), MI, and hypertension	14.9 years	CV mortality (HR = 0.69, 95% CI = 0.45–1.07) and stroke (HR = 0.54, 95% CI = 0.28–1.01) in pet owners among women; stroke mortality in cat owners (HR = 0.22, 95% CI = 0.07–0.68 ^a^)
**Friedmann 2011** [20]	Australia, Canada, New Zealand and US	PR-HAT	Pet	274: 194	14.80%	61.1(9.7)	Survival following MI	2.8 years (median)	Not owning a pet was the only significant independent predictor of mortality (p = 0.036)^a^
**Parker 2010** [10]	Australia	Patients hospitalized with ACS	Pet	204: 220	27.9: 30.9	63.1 (11.9) 67.8 (11.6) ^a^	1-year survival following CAD	1 year	Cat ownership was associated with increased cardiac morbidity and mortality (p = 0.004)^a^
**Gillum 2010** [21]	US	NHANES III, 1988–1994	Pet	3,678: 7,706	56:53	> = 40	All-cause mortality and physical activity	8.5 years	No lower risk of all-cause mortality among those living with canine or feline companions
**Qureshi 2009** [22]	US	NHANES II	Cat	1,015: 2,000	59: 58	47(15): 52(15)	CV mortality	13.4 (3.6) years	CV mortality of MI in past cat owners, RR = 0.63, 95% CI = 0.44–0.88^a^
			Dog	1,932: 843	43: 35	47(14): 55(15)			
**Friedmann 1995** [23]	US and Canada	CAST I, II	Pet	103: 246	14.9	62.8(9.2)	1-year survival following arrhythmia	1 year	1-year survival status was significantly higher in dog owners (p<0.05)^a^
**Friedmann 1980** [24]	US	Patients hospitalized with MI or angina	Pet	53: 39	30.2	-	1-year survival following admission	1 year	1-year survival status was significantly higher in pet owners (p<0.002)^a^

ACS, acute coronary syndrome; ANBP2, Second Australian National Blood Pressure study; SD, standard deviation; CAST, Cardiac Arrhythmia Suppression Trial; CHD, coronary heart disease; CI, confidence interval; CV, cardiovascular; CVD, cardiovascular disease; HR, hazard ratio; HUNT, Norwegian Health Study of Nord-Trondelag; IQR, interquartile range; MI, myocardial infarction; N/A, not applicable; NHANES, National Health and Nutrition Examination Survey; OR, odds ratio; PR-HAT, Psychosocial Responses in the Home Automated External Defibrillator Trial

^a^ *p*<0.05,

^b^ current pet owner

Data were analyzed using the odds ratio (OR) with 95% confidence intervals (CIs) for continuous outcomes. We used software R, Version 1.1.456, a program for statistical computing. We searched the Comprehensive R Archive Network (CRAN) for R packages primarily for meta-analysis and used the *metagen* package for our meta-analysis. Both random and fixed effect models were employed using DerSimonian and Laird’s method. Under the assumption that the true effect size is not the same in all studies, a random effect model was employed[13]. The results derived are presented in Forest plots. Data heterogeneity was quantified using the Cochran Q test and *I*^2^ statistics[14]. Heterogeneity was explained by the subgroup analysis and a meta-regression analysis. Potential publication bias was analyzed with a funnel plot and Egger’s test[15].

## Results

### Description of studies and quality assessment

Fig 1 illustrates the search process. A total of 12 cohort studies were included for our systematic review[8–10, 16–24]. The characteristics of these studies are shown in Table 1. All of the included studies were published between 1990 and 2018. Most of the studies were conducted in North America, Europe, and Australia. Only one was conducted in China[16]. Dogs and cats were the most common pet animals. Most articles did not specify the type of pet ownership, and only one article specified that “pet” included dog, cat, bird, fish, horse, and other[18]. Three publications preferentially discussed dog and cat ownership[16, 19, 22]. A total of 488,986 participants were included; females comprised 52.3% of the participants. The average age of participants was 56.1 years, with those of pet owners and non-pet owners were 54.0±9.2 and 58.2±8.1 years, respectively, without significant difference (*p* = 0.32). Their mean follow-up duration was 8.7±6.3 years.

We assessed the quality of these studies using the Newcastle-Ottawa Scale. The mean score of our included studies was 7, out of the full score 8. In terms of assessing the quality of the selection, if the article focused on the follow-up survival rate of a “selected hospitalized” population with established CVD,[10, 16, 24] zero points were given regarding the representativeness of the exposed cohort. If the article did not report the exclusion criteria, the score indicating that the outcome of interest was not present at the start of the study would be zero[8, 17–19, 21–23]. Most of the studies that we included obtained information about pet ownership using questionnaires; a few used structured interviews or registered pet data. No actual home investigation was mentioned. For these, no point was given for the ascertainment of exposure[17–18, 20, 23–24]. In assessing the comparability of these studies, most of these studies adjusted social economic confounding variables using several different models during survival analysis; as such, only select studies got zero points[20, 23–24]. One study did not specify the actual items of the physiologic and psychosocial variables[23], another study adjusted only for depression status,[20] and a more dated article did not adjust for any variables[24]. When assessing the outcome, the rate of the participants that were lost to follow up was difficult to obtain in secondary surveys[8–10, 16–22], as this was often reported only in the primary report[23–24]. The detailed scores of each study are summarized in S2 Appendix.

### Results of the meta-analysis

**Fig 2:**
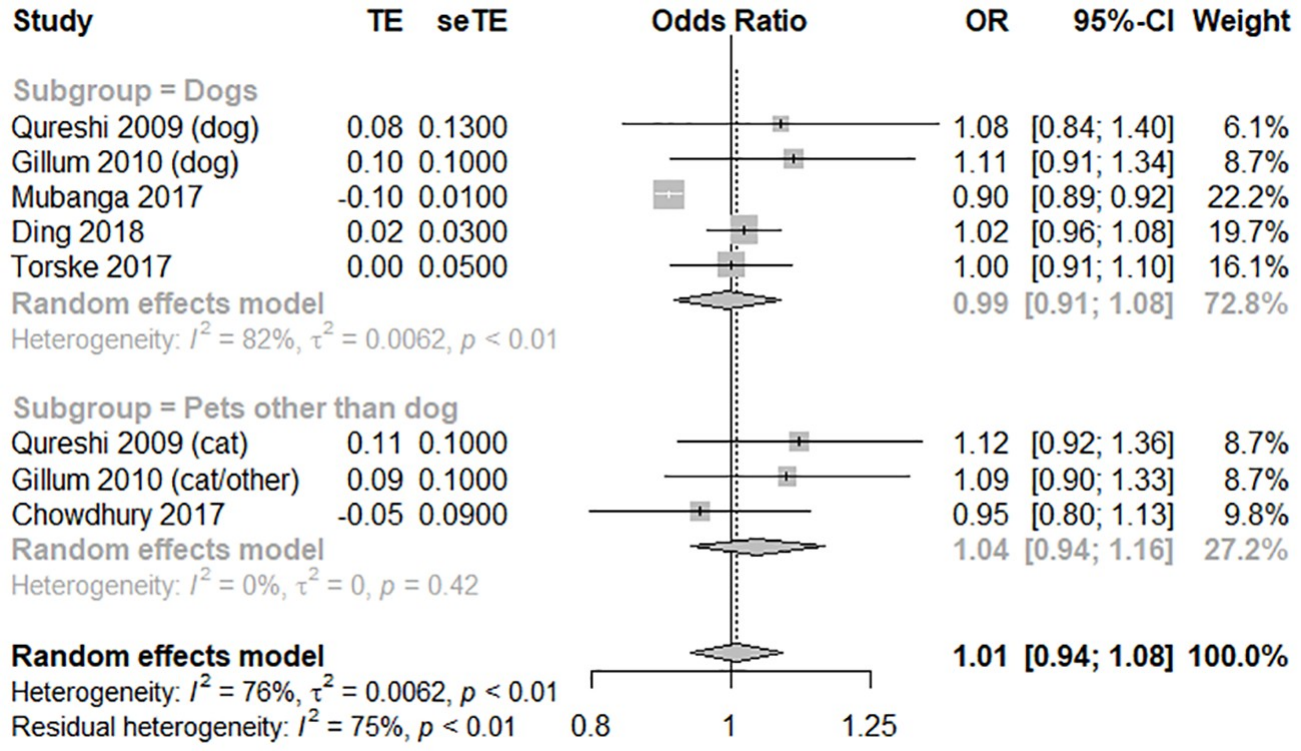
A forest plot of adjusted all-cause mortality, comparing pet owners and non-pet owners, with a subgroup analysis by pet type. CI, confidence interval; OR, odds ratio; SE, standard error; TE, treatment effect.

Twelve cohort studies were included in our systematic review. In terms of adjusted all-cause mortality, six high-quality studies with an average score of 7 were pooled to perform a meta-analysis [8–9,17–18,21–22]. Pet owners did not significantly differ from non-pet owners in terms of adjusted all-cause mortality (OR = 1.01, 95% CI [0.94, 1.08], *I*^*2*^ = 76%). Due to the relatively moderate heterogeneity, we performed a subgroup analysis according to the type of pet animals. Some of the studies included focused solely on dogs, while others accounted for all pets. We therefore defined our subgroups as either “dog owner” or “pet (other than dog) owner.” Heterogeneity improved after performing a subgroup analysis, but no statistical differences were observed in the data. Dog owners were not significantly different from pet (other than dog) owners in terms of adjusted all-cause mortality (OR = 0.99, 95% CI [0.91, 1.08], *I* ^*2*^ = 82%; OR 1.04, 95% CI [0.94, 1.16], *I* ^*2*^ = 0%); the forest plot is shown in Fig 2. To explore the interaction between the different kinds of pets owned, we performed a meta-regression analysis for all of our five outcomes. No multiplicative interactions across animal types were noted (S1A–S1E Fig).The funnel plot was asymmetrical on inspection, and the *p* value of Egger’s test was 0.01, which indicated that a potential publication bias may exist (S2A Fig).

**Fig 3:**
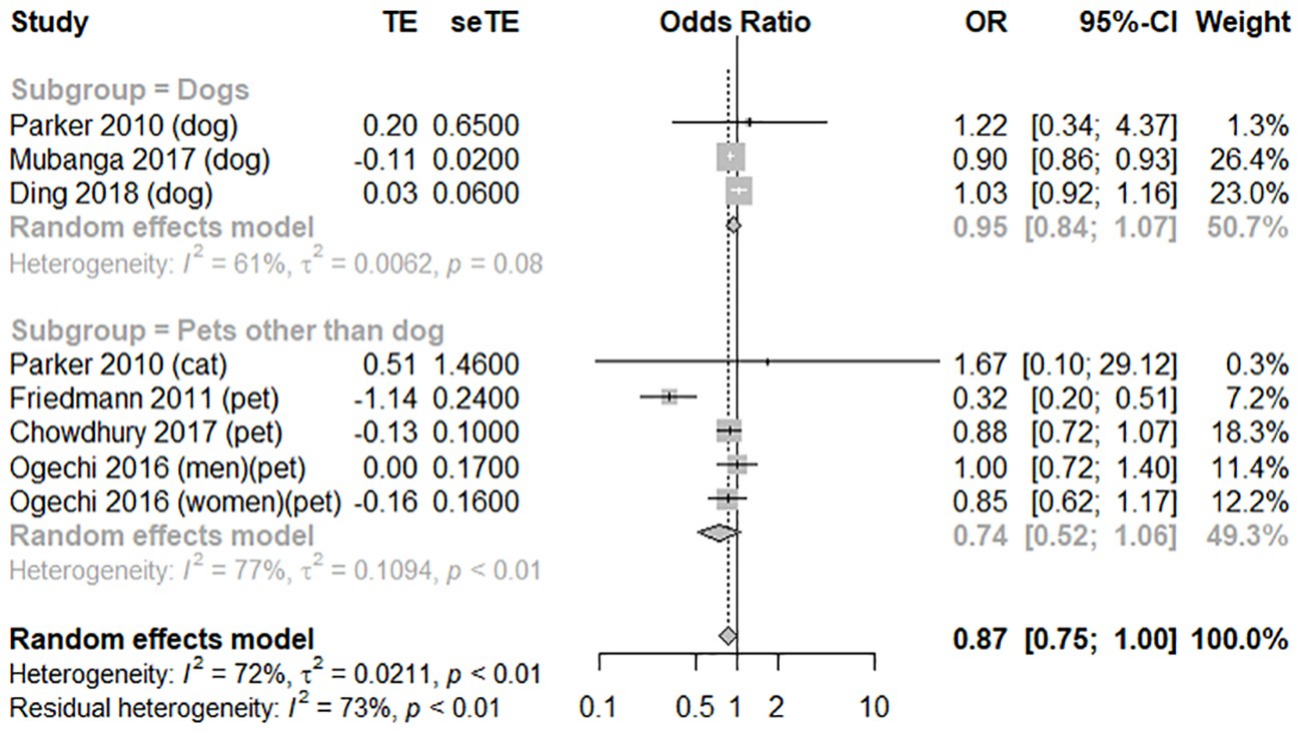
A forest plot of adjusted cardiovascular mortality, comparing pet owners and non-pet owners, with a subgroup analysis by pet type. CI, confidence interval; OR, odds ratio; SE, standard error; TE, treatment effect.

**Fig 4:**
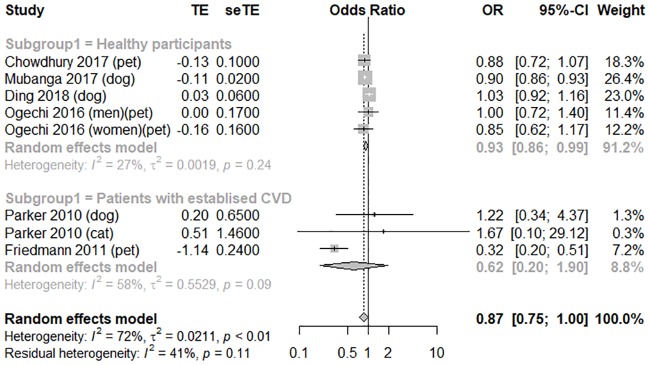
A forest plot of adjusted cardiovascular mortality, comparing pet owners and non-pet owners, with a subgroup analysis by participants health status. CI, confidence interval; OR, odds ratio; SE, standard error; TE, treatment effect.

In terms of adjusted CV mortality, six moderate- to high-quality studies with an average score of 5 were pooled to perform a meta-analysis [8–10,18–20]. Pet owners was statistically borderline differ from non-pet owners in terms of adjusted CV mortality (OR = 0.87, 95% CI [0.75, 1.00], and *I*^*2*^ = 72%). However, based on multiple comparisons, it would not be considered as a protective association. In the subgroup analysis of animal types, dog owners were not significantly different from pet (other than dog) owners in terms of adjusted CV mortality (OR = 0.95, 95% CI [0.84, 1.08], *I* ^*2*^ = 61%; OR 0.74, 95% CI [0.52, 1.06], *I* ^*2*^ = 77%); the forest plot is shown in Fig 3. These studies can be categorized according to two designs: the healthy general population was evaluated using a secondary analysis of a large cohort study that was originally gathered for another study purpose, or a follow-up study on the survival rate of a hospitalized population with established CVD [10, 24]. We therefore performed another subgroup analysis according to the health status of the participants. Compared to the patients with established CVD (OR = 0.62, 95% CI [0.20, 1.90], *I* ^*2*^ = 58%), the general population showed a significant association between pet ownership and lower adjusted CV mortality (OR = 0.93, 95% CI [0.86, 0.99], *I* ^*2*^ = 27%); the forest plot is shown in Fig 4. The funnel plot was symmetrical on inspection, and the *p* value of Egger’s test was 0.73, thus indicating no publication bias (S2B Fig). We performed sensitivity analyses to assess the consistency and credibility of the results by removing studies one by one. In these sensitivity analyses, the pooled findings were changed (S3A–S3H Fig).

**Fig 5:**
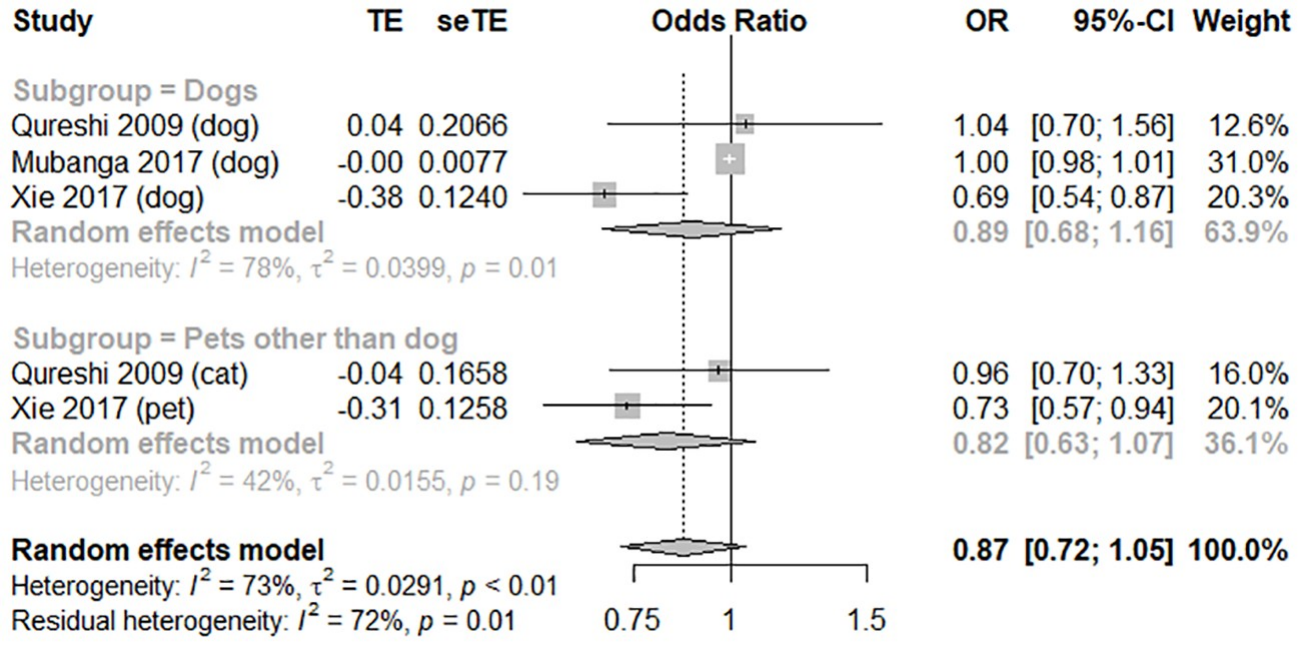
A forest plot showing the risk of cardiovascular disease, comparing pet owners and non-pet owners, with a subgroup analysis by pet type. CI, confidence interval; OR, odds ratio; SE, standard error; TE, treatment effect.

**Fig 6:**
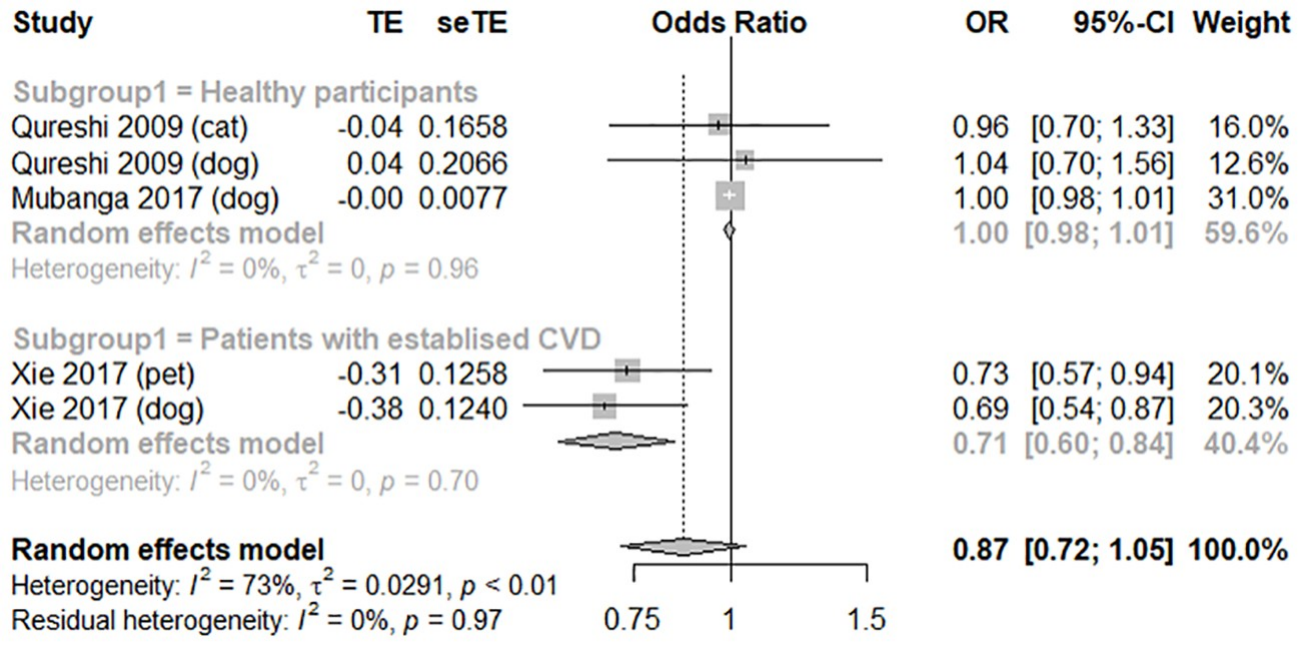
A forest plot showing the risk of cardiovascular disease, comparing pet owners and non-pet owners, with a subgroup analysis by participants health status. CI, confidence interval; OR, odds ratio; SE, standard error; TE, treatment effect.

In terms of the risk of CVD, three high-quality studies with an average score of 7 were pooled to conduct a meta-analysis [9,19,22]. Dog owners did not significantly differ from non-dog owners in terms of CVD risk (OR = 0.89, 95% CI [0.68, 1.16], *I* ^*2*^ = 78%; OR 0.82, 95% CI [0.63, 1.07], *I* ^*2*^ = 42%), as illustrated in the forest plot in Fig 5. Compared to the general population (OR = 1.00, 95% CI [0.98, 1.01, *I* ^*2*^ = 0%), patients with established CVD showed a significant association between pet ownership and lower adjusted CVD risk (OR = 0.71, 95% CI [0.60, 0.84, *I* ^*2*^ = 0%); the forest plot is shown in Fig 6. The funnel plot was symmetrical on inspection, and the *p* value of Egger’s test was 0.20, thus indicating no publication bias (S2C Fig). Sensitivity analyses showed no major changes in the pooled findings (S3I–S3M Fig).

**Fig 7:**
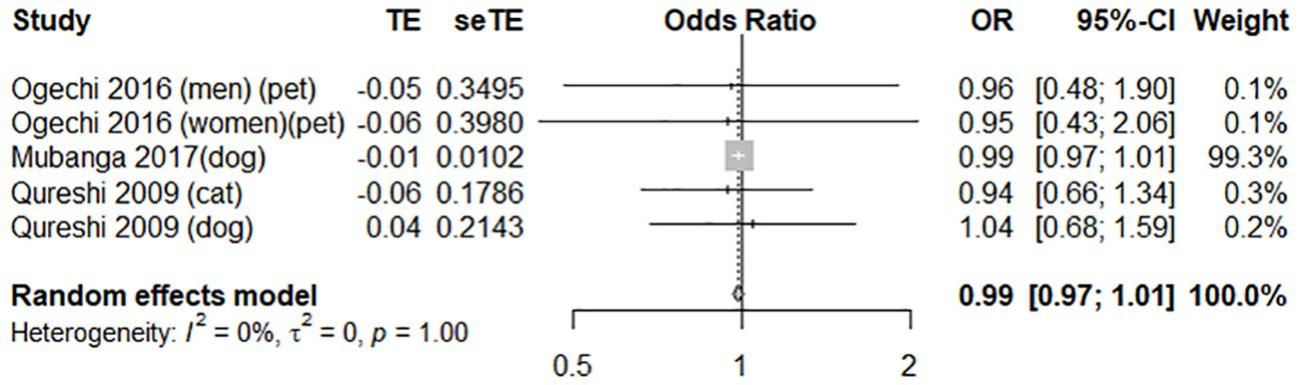
A forest plot of the risk of myocardial infarction between pet owners and non-pet owners. CI, confidence interval; OR, odds ratio; SE, standard error; TE, treatment effect.

In terms of the risk of MI, three high-quality studies with an average score of 7 were pooled in a meta-analysis [9,17,22]. Pet owners did not significantly differ from non-pet owners in terms of MI risk (OR = 0.99, 95% CI [0.97, 1.01], *I* ^*2*^ = 0%). Forest plots of these outcome measures are shown in Fig 7. The funnel plot was symmetrical on inspection, with the *p* value of Egger’s test at 0.67, thus denoting no publication bias (S2D Fig).

**Fig 8:**
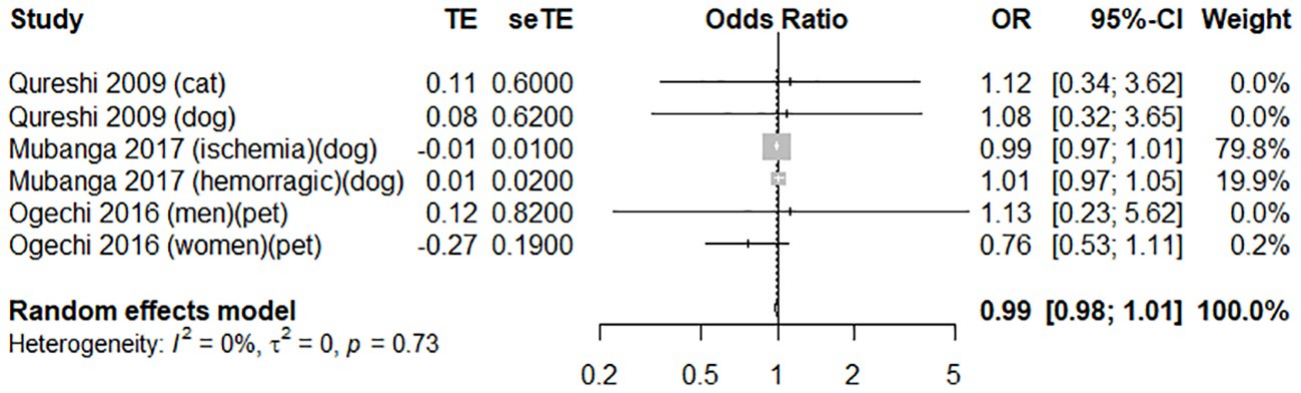
A forest plot comparing the odds ratio of stroke in pet owners and non-pet owners. CI, confidence interval; OR, odds ratio; SE, standard error; TE, treatment effect.

To evaluate the association between pet ownership and the risk of stroke, three high-quality studies with an average score of 7 were pooled to perform a meta-analysis [9,16,22]. Pet owners did not significantly differ from non-pet owners in terms of stroke risk (OR = 0.99, 95% CI [0.98, 1.01], *I* ^*2*^ = 0%); the forest plot is shown in Fig 8. The funnel plot was symmetrical on inspection, and the *p* value of Egger’s test was 0.73, thus indicating no publication bias (S2E Fig).

## Discussion

Our meta-analysis found that pet ownership did not have a significant association with adjusted all-cause or CV mortality or risk of CVD, MI, or stroke. These results were not affected by the type of animal owned. Pet ownership was associated with a lower adjusted CV mortality in the general population relative to CVD patients, and that in patients with established CVD it was associated with significant association with lower adjusted CVD risk relative to the general population. However, sensitivity analyses indicated that these results were not robust, and the meta-regression analysis revealed no multiplicative interaction between animal type and outcome.

Most of the studies we analyzed were conducted in Europe, Australia, and North America, with only one in Asia. Since racial diversity has been found to be associated with pet ownership [25], this could be a confounding factor in our study. The single Asian study in our analysis (from China) found a positive, even dose-sensitive, association of pet ownership with CV outcomes: duration of pet ownership, age at onset of pet ownership, and time spent playing with pets all affected the association [16]. More studies on Asian populations are required to confirm this association. A cohort study performed in Sweden was sufficiently well designed that both a national cohort (almost three million participants) and a twin cohort were analyzed, and this contributed substantially to our findings [9].

The funnel plots for the pooled data suggested the existence of publication bias with respect to all-cause mortality data. However, these plots may not be reliable, due to the small number of studies included.

Our subgroup analysis was conducted according to the health status of the participants. Both the patients with established CVD and the general population derived benefits from pet ownership with respect to different outcomes, and heterogeneity was greatly reduced in the subgroups. In the studies used, the definitions of CVD, MI, and stroke were mostly based on the International Classification of Diseases (ICD) system, 9^th^ or 10^th^ Revision (ICD-9 or ICD-10). Most articles used the same definition of CVD as we did, which included all stroke, MI, and heart failure events. There were a few minor discrepancies between studies: one paper differed in its definition of CVD [16], though the definition of MI was more consistent, with all three studies using the same ICD codes [9, 16, 22]; and five surveyed the survival rate following a CV event. The results of these studies also varied substantially. One indicated a strong but widely varying negative association of cat ownership on CV-related deaths [22], while another found a strong positive association of pet ownership on the risk of CVD [16]. Indeed, the sensitivity analyses of adjusted CV mortality revealed differences in the pooled findings from those of most of the studies individually. The sample size varied widely across studies, from hundreds to millions, so the weight of each study varied from 0.3% to 26.4%. For the larger studies (> 10% weight), the results were not consistent, and removing just one large study could change the findings completely. In the sensitivity analyses of the risk of CVD, most results suggested that pet ownership was beneficial. However, when we removed one large study [9], with 31% weight and an unfavorable result, the pooled result changed. Whether pet ownership has more positive association with general population or CVD patient, the results from the presently available large studies may not be robust, and more large studies focusing on pet ownership with CV outcomes, comparing groups with established CVD with the general population, are warranted.

We found that pet ownership was not significantly associated with adjusted CV or all-cause mortality. However, numerous factors were associated with mortality. We tried to extract the outcomes controlled according to various socioeconomic variables, but the factors controlled for varied among the studies. Furthermore, pet owners differ from non-pet owners in many ways, and these differences may contribute to the possible CV benefits suggested by the raw data, although it may be possible to control for these benefits if we could adjust the data for all the relevant socioeconomic factors.

We also performed a subgroup analysis according to type of pet. Few of the studies investigated the association between animal type or timing of pet ownership and CV, The profile of pet owners in general may explain this finding. Pet owners tended to be younger, female, married, live in the countryside, have family, get more exercise, be better educated, and be employed [26–27]. These factors contribute to a higher self-health awareness, which may be related to more frequent disease screening, the early detection of diseases, and a greater chance of accessing better medical care and support, possibly leading to a more favorable health outcome. A range of socioeconomic health inequalities, including health care provider selection, relative resource deprivation, and the accumulation of socially patterned exposures throughout life also influence health outcomes [28–32].

The benefits of having companion animals not only involves both psychological and social aspects related to the characteristics of pet ownership, but also involve biological effects. Human–animal interactions are beneficial for social interaction, fear and anxiety, and mental and physical health, and stress-related parameters such as cortisol, blood pressure, and heart rate, tend to be more stable in pet owners [33]. In both dogs and their owners, oxytocin and cortisol levels are associated with their interactions and behaviors [34].

Although not all of our findings were statistically significant, pet ownership should not be dismissed as non-relevant for health, since the meta-analysis employed a comprehensive search strategy, and the studies included had large sample sizes and long-term follow-up data. However, this study has several limitations. First, there were no randomized control trials included. The objective of such a study and the target population would render an experimental setup difficult. We therefore tried to control for confounding factors by extracting adjusted data, to obtain more accurate results, but as mentioned above, the adjustments used varied among these observational studies. In addition, if the non-pet owners were systematically different from the pet owners, such adjustment may not have been sufficient to correct the sampling design. Second, most of the studies included were secondary surveys. Primary studies would have been more convincing, but no such data are currently available. Nevertheless, all the studies were of good quality, which minimized this problem. Third, the types of animals kept as pets were not detailed in the studies analyzed. More studies comparing the associations of pet ownership on CV outcomes, according to the type of pet kept, should therefore be conducted.

## Conclusion

This meta-analysis found no association between pet ownership and all-cause mortality or the CV outcomes of CVD risk, adjusted CV mortality, or risk of MI or stroke. Despite this, subgroup analysis showed an association between pet ownership and was associated with a lower CVD mortality in the general population, and between pet ownership and adjusted CVD risk in patients with established CVD. However, the results were not robust, and the type of pet owned did not appear to affect the association. More studies on the association of pet ownership with CV outcomes are needed, with additional focus on establishing socioeconomic profiles and the details of pet ownership.

## Supporting information

S1 Table PRISMA checklist. (PDF)

S2 Table Sensitivity test of adjusted cardiovascular mortality. (PDF)

S3 Table Sensitivity test of adjusted risk of CVD. (PDF)

S1 Appendix Search strategy. (PDF)

S2 Appendix Detailed Newcastle-Ottawa Scale of each included study. (PDF)

S1 Fig (A-E) Meta-regression between pet type and outcomes. (PDF)

S2 Fig (A-E) The funnel plots of outcomes. (PDF)

S3 Fig (A-M). Forest plot of sensitivity analyses. (PDF)

S1 Dataset (PDF)
