# Supplementary material for: A modest protective association between pet ownership and cardiovascular diseases: A systematic review and meta-analysis
Source: PLoS One. 2019 May 3;14(5):e0216231. doi: 10.1371/journal.pone.0216231 (PMC6499429; doi:10.1371/journal.pone.0216231)
Supplement: S3 Table — (PDF) [file pone.0216231.s003.pdf]

S3 Table. Sensitivity test of adjusted risk of CVD

Exclude article	Healthy participants		Patients with established CVD		Total result	
	OR	95% CI	OR	95% CI	OR	95% CI
None	**=1**	**-**	**<1**	**+**	**<1**	**-**
Qureshi 2009 (cat)	=1	-	<1	+	<1	-
Qureshi 2009 (dog)	=1	-	<1	+	<1	-
Mubanga 2017 (dog)	<1	-	<1	+	<1	+
Xie 2017 (pet)	=1	-	<1	+	<1	-
Xie 2017 (dog)	=1	-	<1	+	<1	-

+: significant, -: non-significant
