# Supplementary material for: A modest protective association between pet ownership and cardiovascular diseases: A systematic review and meta-analysis
Source: PLoS One. 2019 May 3;14(5):e0216231. doi: 10.1371/journal.pone.0216231 (PMC6499429; doi:10.1371/journal.pone.0216231)
Supplement: S1 Appendix — (PDF) [file pone.0216231.s004.pdf]

S1 Appendix. Search strategy.

Data base	Items found	Searching date	note
PubMed	1288	20180831	Including Medline /PubMed
EMBASE	2281	20180831	OVID platform
CINAHL	40	20180831	EBSCO platform
Cochrane CDSR	5	20180831	Cochrane Database of Systematic Reviews (CDSR)
Cochrane CENTRAL	145	20180831	Cochrane Central Register of Controlled Trials (CENTRAL)
PerioPath	0	20180831	The PerioPath：Index to Taiwan Periodical Literature System
others	21		
Original total papers	3780		
de-duplicate by EndNote	443		Duplicated defined by same publication year and title
screen by title/abstract	3316		

S1 PICO.

PICO	MeSH term + EMTree term + free textword
Population	(pets OR pet OR dogs OR dog OR cats OR cat) AND (ownership OR owner OR owning OR owned OR companion)
	pet*-dog* OR dog walking OR pet*-cat* OR pet*-animal* OR bonding, Human-Pet
outcome	Mortality OR Mortalities OR fatalityOR death rate OR Cause of Death OR survival OR Cardiovascular Diseases OR CVD OR Heart Diseases OR Cardiac Disease* OR Coronary Disease OR Myocardial Ischemia OR heart Ischemia OR heart attact OR heart attack OR Myocardial Infarction OR Stroke OR cardiovascular risk

PubMed

20180829

S1 . Recent queries in pubmed.

Search	Query	Items found	Time
#41	#20 AND #40	1288	04:15:17
#40	#27 OR #39	3382509	04:02:31
#39	#28 OR #29 OR #30 OR #31 OR #32 OR #33 OR #34 OR #35 OR #36 OR #37 OR #38	2465985	04:02:22
#38	Search cardiovascular risk[Title/Abstract]	58899	04:01:29
#37	Search Stroke	294841	03:59:48
#36	Search Myocardial Infarction	232902	03:59:00
#35	Search heart attack	239886	03:58:08
#34	Search heart Ischemia	75291	03:57:27
#33	Search Myocardial Ischemia	458069	03:57:13
#32	Search Coronary Disease	304064	03:55:52
#31	Search Cardiac Disease*	17652	03:54:45
#30	Search Heart Diseases	1134446	03:54:20
#29	Search CVD[Title/Abstract]	29361	03:53:36
#28	Search Cardiovascular Diseases	2270280	03:53:21
#27	#24 OR #25 OR #26	1235385	03:50:52
#26	Search Survival	1767180	03:50:21
#25	Search Cause of Death	150588	03:48:02
#24	Search death rate	1165175	03:47:17
#23	Search fatality	16211	03:47:02
#22	Search Mortalities	1121788	03:46:43
#21	Search Mortality	1117996	03:46:30
#20	#14 OR #15 OR #16 OR #17 OR #18 OR #19	13189	03:45:56

#19	Search bonding, Human-Pet[mesh]	1751	03:45:18
#18	Search pet*-animal*[Title/Abstract]	326	03:44:22
#17	Search pet*-cat*[Title/Abstract]	370	03:43:51
#16	Search dog walking[Title/Abstract]	112	03:43:31
#15	Search pet*-dog*[Title/Abstract]	916	03:43:11
#14	Search #7 AND #13	10676	03:42:48
#13	Search #8 OR #9 OR #10 OR #11 OR #12 OR #13	85068	03:42:40
#12	Search companion	43089	03:42:23
#11	Search owned	8961	03:42:08
#10	Search owning	1650	03:42:01
#9	Search owner	4374	03:41:50
#8	Search ownership	30178	03:41:42
#7	Search #1 OR #2 OR #3 OR #4 OR #5 OR #6	599802	03:41:26
#6	Search cat	117779	03:40:25
#5	Search cats	140951	03:40:13
#4	Search dog	344268	03:39:54
#3	Search dogs	332213	03:39:38
#2	Search pet	90868	03:39:21
#1	Search pets	6598	03:39:06

EMBASE_Ovid

20180831 **S1 Recent queries in EMBASE_Ovid.**

# ▲	Searches	Results
1	exp pet animal/	6281
2	(pet or pets or dog or dogs or cat or cats or animal*).mp. [mp=title, abstract, heading word, drug trade name, original title, device manufacturer, drug manufacturer, device trade name, keyword, floating subheading word, candidate term word]	5532965
3	1 or 2	5532965
4	(ownership or owner or owning or owned or housed).mp. [mp=title, abstract, heading word, drug trade name, original title, device manufacturer, drug manufacturer, device trade name, keyword, floating subheading word, candidate term word]	53302
5	3 and 4	23719
6	exp human-animal bond/	206
7	dog walking.mp.	118
8	anthrozoology.mp.	5
9	5 or 6 or 7 or 8	23933
10	mortality/ or all cause mortality/ or mortality rate/ or mortality risk/ or cardiovascular mortality/	746349
11	"cause of death"/	102269

12	(mortality or mortalities or fatality or death rate or deathcause or survival).mp. [mp=title, abstract, heading word, drug trade name, original title, device manufacturer, drug manufacturer, device trade name, keyword, floating subheading word, candidate term word]	2524453
13	cardiovascular disease/ or cardiovascular risk/	369574
14	heart disease/	98499
15	coronary artery disease/	173788
16	exp heart muscle ischemia/	87470
17	acute heart infarction/ or ischemic heart disease/ or heart infarction/	397694
18	myocardial infraction.mp. [mp=title, abstract, heading word, drug trade name, original title, device manufacturer, drug manufacturer, device trade name, keyword, floating subheading word, candidate term word]	906
19	stroke.mp. [mp=title, abstract, heading word, drug trade name, original title, device manufacturer, drug manufacturer, device trade name, keyword, floating subheading word, candidate term word]	383208
20	10 or 11 or 12 or 13 or 14 or 15 or 16 or 17 or 18 or 19	3506817
23	9 and 20	2281

CINAHL

20180831 **S1 Recent queries in CINAHL.**

Search ID no.	Query	Items found
S30	S18 AND S29	40
S29	S19 OR S20 OR S21 OR S22 OR S23 S24 OR S25 OR S26 OR S27 OR S28	149184
S28	(MH "Stroke") OR "stroke"	67652
S27	(MH "Myocardial Infarction") OR "myocardial infarction" OR (MH "Myocardial Diseases")	38854
S26	(MH "Heart Diseases") OR "heart diseases" OR (MH "Myocardial Ischemia") OR (MH "Coronary Disease")	29676
S25	TI CVD	606
S24	AB CVD	4891
S23	(MH "Cardiovascular Diseases") OR "cardiovascular diseases"	29143
S22	S20 OR S21 OR S22	146399
S21	(MH "Cause of Death") OR "cause of death"	12554
S20	"death rate"	1223
S19	(MH "Mortality") OR "mortality"	140947
S18	S10 OR S17	2165
S17	S11 OR S12 OR S13 OR S14 OR S15 OR S16	2053
S16	(MH "Human-Pet Bonding")	470

S15	dog walking	146
S14	pet animals	1368
S13	pet-cat	17
S12	pet-dog	56
S11	(MH "Service Animals")	387
S10	S4 AND S9	338
S9	S5 OR S6 OR S7 OR S8	6419
S8	"owned"	1436
S7	"companion"	1543
S6	"owner"	543
S5	"ownership"	3176
S4	S1 OR S2 OR S3	8355
S3	(MH "Cats") OR "cats"	1600
S2	(MH "Dogs") OR "dogs"	5707
S1	(MH "Pets") OR "pets"	2122

S1 Recent queries in Cochrane.

Search | Search manager | Medical terms (MeSH)

Save this search | View saved searches | ? Search help

View fewer lines | Print

#1	MeSH descriptor: [Bonding, Human-Pet] explode all trees	MeSH	24
#2	MeSH descriptor: [Pets] explode all trees	MeSH	12
#3	(pet or pets or dog or dogs or cat or cats):ti,ab,kw	S	7603
#4	MeSH descriptor: [Ownership] explode all trees	MeSH	104
#5	(ownership or owner or owing or owned or companion or companionship):ti,ab,kw	S	3969
#6	#2 or #3		7603
#7	#4 or #5		4039
#8	#6 and #7		137
#9	#8 or #1		154
#10	MeSH descriptor: [Mortality] explode all trees	MeSH	12276
#11	MeSH descriptor: [Survival] explode all trees	MeSH	128
#12	(mortality or mortalities or death or died svrvival):ti,ab,kw	S	96218
#13	MeSH descriptor: [Cardiovascular Diseases] explode all trees	MeSH	93661
#14	(cardio* or CVD or heart or coronary or myocardial of stroke or ischem* or infarction):ti,ab,kw	S	185212
#15	#10 or #11 or #12 or #13 or #14		274562
#16	#9 and #15		27
#17	Manually type a search term here or click on the S (Search Wizard) or MeSH button to compose one	S MeSH	N/A

Clear all

Highlight orphan lines
