# Supplementary material for: A modest protective association between pet ownership and cardiovascular diseases: A systematic review and meta-analysis
Source: PLoS One. 2019 May 3;14(5):e0216231. doi: 10.1371/journal.pone.0216231 (PMC6499429; doi:10.1371/journal.pone.0216231)
Supplement: S2 Appendix — (PDF) [file pone.0216231.s005.pdf]

S2 Table A. Detailed Newcastle-Ottawa Scale of each included cohort study.

	Selection				Comparability		Outcome			
Study	Representativeness of exposed cohort	Selection of non-exposed cohort	Ascertainment of exposure	Demonstration that outcome of interest was not present at start of study	Adjust for the most important risk factors	Adjust for other risk factors	Assessment of outcome	Follow-up length	Loss to follow-up rate	**Total quality score**
Ding 2018	1	1	1	0	1	1	1	1	0	7
Torske 2017	1	1	0	0	1	1	1	1	0	6
Mubanga 2017	1	1	1	1	1	1	1	1	0	8
Chowdhury 2017	1	1	0	0	1	1	1	1	0	6
Ogechi 2016	1	1	1	0	1	1	1	1	0	7
Friedman 2011	1	1	0	1	0	1	0	1	0	5
Parker 2010	0	1	1	1	1	1	1	1	0	7
Guillum 2010	1	1	1	0	1	1	1	1	0	7
Qureshi 2009	1	1	1	0	1	1	1	1	0	7
Friedman 1995	1	1	0	0	0	1	1	1	1	6
Friedman 1980	0	1	0	1	0	0	1	1	1	5

S2 Table B. Detailed Newcastle-Ottawa Scale of each included cross-sectional study.

	Selection				Comparability	Outcome		
Study	Representativeness of the sample	Sample size	Non-respondents	Ascertainment of the exposure (risk factor)	Confounding factors controlled	Assessment of outcome	Statistical test	**Total quality score**
Xie 2017	1	1	1	1	1	1	1	**7**

The quality of included studies was assessed by the Newcastle Ottawa scale. A study can be awarded a maximum of one star for each
numbered item within the Selection and Outcome categories and a maximum of two stars for Comparability.
Selection:
1) Representativeness of exposed cohort: 1, study population truly or somewhat representative of a community/ population-based study; 0, study population was sampled from a special population, that is, population from a company, hospital patients, data from the health insurance company or health examination organization, nurses.
2) Selection of non-exposed cohort: 1, drawn from the same community as the exposed cohort.
3) Ascertainment of exposure: 1, Validation of pets use with secure record; 0, no specific pets use validation method.
4) Demonstration that outcome was not present at start of study: 1, exclusion of participants with a history of severe ventricular arrhythmia or sudden cardiac arrest at the beginning of the study.
Comparability: 1) 1, whether a study adjusted for the most important factors deliberately; 1, whether a study adjusted for other important risk factors.
Outcome:
1) Assessment of outcome: 1, cardiovascular events were confirmed by medical records or record linkage; 0, self-reported.
2) Was follow-up long enough for outcomes to occur: 1, duration of follow-up >= 5 year; 0, if duration of follow-up < 5 year.
3) Loss to follow-up rate: 1, complete follow-up or loss to follow up rate <=20 %; 0, follow-up rate < 80% or no description of those
lost.

NEWCASTLE - OTTAWA QUALITY ASSESSMENT SCALE

COHORT STUDIES Ding 2018

Selection

1) <u>Representativeness of the exposed cohort</u>

a) truly representative of the average __population_ in the community *

Adult participants (aged ≥16 years) were drawn from the Health Survey for England (HSE) 1995, 1996, 1997, 2001, 2002, and 2004. HSE is a population-based study running continuously in annual thematic cycles and has been linked to the National Death Registry.10

b) somewhat representative of the average _____________ in the community *

c) selected group of users eg nurses, volunteers

d) no description of the derivation of the cohort

2) <u>Selection of the non exposed cohort</u>

a) drawn from the same community as the exposed cohort *

b) drawn from a different source

c) no description of the derivation of the non-exposed cohort

3) Ascertainment of exposure

a) secure record (eg surgical records) *

b) structured interview *the individual-level interviewer- administered questionnaire.

c) written self report

d) no description

4) Demonstration that outcome of interest was not present at start of study

a) yes *

b) no, the secondary survey did not mention the exclusion criteria, if other high risk of sudden death or mortality exist in the start

Comparability

1) Comparability of cohorts on the basis of the design or analysis

a) study controls for _age and sex_(modle 1) *

b) study controls for any additional various sociodemographic and lifestyle variables*

Model 2: adjusted for variables in Model 1 + marital status, social class, employment status, educational attainment, and living situation;

Model 3: adjusted for variables in Model 2 + alcohol consumption, smoking status, and long-standing illness.

Outcome

1) Assessment of outcome

a) independent blind assessment *

b) record linkage * linked to the National Death Registry

c) self-report

d) no description

2) Was follow-up long enough for outcomes to occur

a) yes (select an adequate follow up period for outcome of interest) *11.5(3.8) years

b) no

3) Adequacy of follow up of cohorts

a) complete follow up - all subjects accounted for *

b) subjects lost to follow up unlikely to introduce bias - small number lost

c) follow up rate < ____% (select an adequate %) and no description of those lost

d) no statement

NEWCASTLE - OTTAWA QUALITY ASSESSMENT SCALE

COHORT STUDIES Torske 2017

Note: A study can be awarded a maximum of one star for each numbered item within the Selection and Outcome categories. A maximum of two stars can be given for Comparability

Selection

1) Representativeness of the exposed cohort

a) truly representative of the average __population_ in the community *

We used data from the second wave of the Nord-Trondelag HUNT Study (HUNT2, 1995±1997) in our study. All residents of Nord-Trondelag County, Norway, aged 20 and above, were invited by mail.

b) somewhat representative of the average _____________ in the community *

c) selected group of users eg nurses, volunteers

d) no description of the derivation of the cohort

2) Selection of the non exposed cohort

a) drawn from the same community as the exposed cohort *

b) drawn from a different source

c) no description of the derivation of the non exposed cohort

3) Ascertainment of exposure

a) secure record (eg surgical records) *

b) structured interview *

c) written self-report (questionnaire)

d) no description

4) Demonstration that outcome of interest was not present at start of study

a) yes *

b) no, the secondary survey did not mention the exclusion criteria, if other high risk of sudden death or mortality exist in the start

Comparability

1) Comparability of cohorts on the basis of the design or analysis

a) study controls for _age and sex__(modle 1) *

b) study controls for any additional various sociodemographic and lifestyle variables*

Model 2: adjusted for variables in Model 1 +education and marital status

Model 3: adjusted for variables in Model 2 + anxiety and depression scores, BMI, physical

activity levels and smoking

Outcome

1) Assessment of outcome

a) independent blind assessment *

b) record linkage *The HUNT database is regularly updated through the National Registry on dates of death and emigration of HUNT participants

c) self report

d) no description

2) Was follow-up long enough for outcomes to occur

a) yes (select an adequate follow up period for outcome of interest) *

The median follow-up time was 18.5 years and the maximum follow-up time was 19.7 years.

b) no

3) Adequacy of follow up of cohorts

NEWCASTLE - OTTAWA QUALITY ASSESSMENT SCALE

COHORT STUDIES Mubanga 2017

Note: A study can be awarded a maximum of one star for each numbered item within the Selection and Outcome categories. A maximum of two stars can be given for Comparability

Selection

1) Representativeness of the exposed cohort

a) truly representative of the average __population_ in the community *

Study population – National Cohort. All Swedish residents aged 40 to 80 years

b) somewhat representative of the average __twin population___________ in the community *

Study population –the Swedish Twin Register (STR). The STR, initiated in 1958, is a longitudinal study of the vast majority of twins born in Sweden after 1886

c) selected group of users eg nurses, volunteers

d) no description of the derivation of the cohort

2) Selection of the non exposed cohort

a) drawn from the same community as the exposed cohort *

b) drawn from a different source

c) no description of the derivation of the non-exposed cohort

3) Ascertainment of exposure

a) secure record (eg surgical records) *

Since January 1, 2001, it has been a statutory requirement that every dog in Sweden has a unique identifier (ear tattoo or subcutaneous chip) registered at the Swedish Board of Agriculture. In addition, the Swedish Kennel Club has registered all dogs with a certified pedigree with complete information on owner's personal identity number since 2001.

In 2012, an estimated 83% (95% confidence interval, CI, 78–87%) dogs were registered

in at least one of these two register

Dog ownership was defined as periods registered or having a partner registered as a dog owner in either of the two dog registers

b) structured interview *

c) written self-report (questionnaire)

d) no description

4) Demonstration that outcome of interest was not present at start of study

a) yes *

Individuals with inpatient visits (n = 387,571 in national cohort and n = 3,163 in twin cohort) between January 1, 1987 to December 31, 2000 for CVD (ICD-9 codes 390–459 or ICD-10 I00-I99; main or secondary diagnosis) and/or had a coronary artery bypass grafting or percutaneous coronary artery intervention (Nordic surgical procedure codes FNA, FNC and FNG) were excluded from the study.

According to the exclusion criteria, they tried to excluded established CVD from the start.

b) no

Comparability

1) Comparability of cohorts on the basis of the design or analysis

a) study controls for _ sex__(modle 1) *

b) study controls for any additional various sociodemographic and lifestyle variables*

Model 2: adjusted for sex, marital status, presence of children in the home, population density, area of residence, region of birth, income and latitude

Outcome

1) Assessment of outcome

a) independent blind assessment *

b) record linkage *

death data from the Cause of Death Register

the main diagnosis in inpatient and outpatient care and underlying cause of death were used to define four incident disease outcomes: (1) acute myocardial infarction (ICD-10 I21); (2) heart failure (ICD-10 I50); (3) ischemic stroke (ICD-10 I63) and (4) hemorrhagic stroke (ICD-10 I60-I62).

c) self report

d) no description

2) Was follow-up long enough for outcomes to occur

a) yes (select an adequate follow up period for outcome of interest) *12 years

b) no

3) Adequacy of follow up of cohorts

a) complete follow up - all subjects accounted for *

b) subjects lost to follow up unlikely to introduce bias - small number lost - >   *

c) follow up rate < ____% (select an adequate %) and no description of those lost

d) no statement

NEWCASTLE - OTTAWA QUALITY ASSESSMENT SCALE

COHORT STUDIES Chowdhury 2017

Note: A study can be awarded a maximum of one star for each numbered item within the Selection and Outcome categories. A maximum of two stars can be given for Comparability

Selection

1) Representativeness of the exposed cohort

a) truly representative of the average __population_ in the community *

6083 participants aged between 65 and 84 years were enrolled between 1995 and 1997 from five states of Australia with an untreated BP of at least 160mmHg SBP and/or at least 90mmHg DBP

before randomization.c) selected group of users eg nurses, volunteers

d) no description of the derivation of the cohort

2) Selection of the non exposed cohort

a) drawn from the same community as the exposed cohort *

b) drawn from a different source

c) no description of the derivation of the non-exposed cohort

3) Ascertainment of exposure

a) secure record (eg surgical records) *

b) structured interview *

c) written self- report questionnaire

d) no description

4) Demonstration that outcome of interest was not present at start of study

a) yes *

b) no, the secondary survey did not mention the exclusion criteria, if other high risk of sudden death or mortality exist in the start

Comparability

1) Comparability of cohorts on the basis of the design or analysis

a) study controls for _ sex_and age__(modle 2) *

b) study controls for any additional various sociodemographic and lifestyle variables*

Model 3: 2+ education, marital status, BP, TC, HDL, DM HX, smoking, BMI, GFR, PA

Model 4: 3+randomized treatment group (ACE-I/diuretic) and BP

Outcome

1) Assessment of outcome

a) independent blind assessment *

b) record linkage *

survival information was determined by linkage to the Australian Institute of Health

and Welfare National Death Index (death registry). International Classification of Disease version 10 coding was used to identify cause of death

c) self-report

d) no description

2) Was follow-up long enough for outcomes to occur

a) yes (select an adequate follow up period for outcome of interest) *

a median of 10.9 years (interquartile range: 10.2–11.4) until data were censored at 31 October 2009.

b) no

3) Adequacy of follow up of cohorts

d) no statement

NEWCASTLE - OTTAWA QUALITY ASSESSMENT SCALE

COHORT STUDIES Ogechi 2016

Note: A study can be awarded a maximum of one star for each numbered item within the Selection and Outcome categories. A maximum of two stars can be given for Comparability

Selection

1) Representativeness of the exposed cohort

a) truly representative of the average __population_ in the community *

The NHANES III consisted of a nationwide probability sample of non-institutionalized civilians.before randomization.

c) selected group of users eg nurses, volunteers

d) no description of the derivation of the cohort

2) Selection of the non exposed cohort

a) drawn from the same community as the exposed cohort *

b) drawn from a different source

c) no description of the derivation of the non-exposed cohort

3) Ascertainment of exposure

a) secure record (eg surgical records) *

b) structured interview *household interview,

c) written self-report (questionnaire)

d) no description

4) Demonstration that outcome of interest was not present at start of study

a) yes *

b) no, the secondary survey did not mention the exclusion criteria, if other high risk of sudden death or mortality exist in the start

Comparability

1) Comparability of cohorts on the basis of the design or analysis

a) study controls for _ sex_and age_(modle 2) *

b) study controls for any additional various sociodemographic and lifestyle variables*

Adjusted for age, race, family income, education attainment, alcohol drinking, cigarette smoking, marital status, body mass index, physical activity level assessed at baseline survey

Outcome

1) Assessment of outcome

a) independent blind assessment *

b) record linkage *

ICD 9 or 10

c) self-report

d) no description

2) Was follow-up long enough for outcomes to occur

a) yes (select an adequate follow up period for outcome of interest) *

The average follow-up time was 14.9 years with a range of 0.7–18.1 years.

b) no

3) Adequacy of follow up of cohorts

c) follow up rate < ____% (select an adequate %) and no description of those lost

d) no statement

NEWCASTLE - OTTAWA QUALITY ASSESSMENT SCALE

COHORT STUDIES Friedman 2011

Note: A study can be awarded a maximum of one star for each numbered item within the Selection and Outcome categories. A maximum of two stars can be given for Comparability

Selection

1) Representativeness of the exposed cohort

a) truly representative of the average __population_ in the community *

b) somewhat representative of the average __post MI patient_in the community *

Patients were eligible for recruitment into HAT if they had an anterior myocardial infarction a minimum of 6 months prior to recruitment at 30 sites in Australia (12), Canada (5), New Zealand (2), and the United States (11)

c) selected group of users eg nurses, volunteers

d) no description of the derivation of the cohort

2) Selection of the non exposed cohort

a) drawn from the same community as the exposed cohort *

b) drawn from a different source

c) no description of the derivation of the non-exposed cohort

3) Ascertainment of exposure

a) secure record (eg surgical records) *

b) structured interview *

c) written self-report Pet Ownership Questionnaire

d) no description

4) Demonstration that outcome of interest was not present at start of study

a) yes * post-MI patients who have already survived at least 6 months. Complete HAT inclusion and exclusion criteria and recruitment methods have been published elsewhere (Bardy et al., 2008; Bardy et al., 2008).

b) no

Comparability

1) Comparability of cohorts on the basis of the design or analysis

a) study controls for the most important factor) *

b) study controls for _depression and its interaction with pet ownerships*

Outcome

1) Assessment of outcome

a) independent blind assessment *

b) record linkage *

c) self report

d) no description

2) Was follow-up long enough for outcomes to occur

a) yes (select an adequate follow up period for outcome of interest) Seventeen post MI patients died during a follow-up of 0 to 4.2 years, with a median follow up of 2.8 years.

b) no

3) Adequacy of follow up of cohorts

a) complete follow up - all subjects accounted for *

b) subjects lost to follow up unlikely to introduce bias - small number lost - > ____ % (select an adequate %) follow up, or description

d) no statement

NEWCASTLE - OTTAWA QUALITY ASSESSMENT SCALE

COHORT STUDIES Parker 2010

Note: A study can be awarded a maximum of one star for each numbered item within the Selection and Outcome categories. A maximum of two stars can be given for Comparability

Selection

1) Representativeness of the exposed cohort

a) truly representative of the average __population_ in the community *

b) somewhat representative of the average __ _in the community *

c) selected group of users eg nurses, volunteers, hospitalized

Participants were recruited over a 2-year period from adult patients admitted to the Cardiology unit at a large Sydney teaching hospital (the Prince of Wales Hospital) from Sydney and surrounding districts with an ACS

d) no description of the derivation of the cohort

2) Selection of the non exposed cohort

a) drawn from the same community as the exposed cohort *

b) drawn from a different source

c) no description of the derivation of the non exposed cohort

3) Ascertainment of exposure

a) secure record (eg surgical records) *

b) structured interview *participants were asked if they had a pet in their household

c) written self-report

d) no description

4) Demonstration that outcome of interest was not present at start of study

a) yes *

Potential participants were excluded if they were non-English speaking, cognitively impaired, had significant hearing or visual impairment, had a high mortality risk from other medical conditions (e.g. metastatic cancer) or were admitted for a scheduled coronary artery bypass graft.

The study is aimed to perform on high risk sudden death patients, restrict the past VT history is difficult. They had tried to exclude high mortality risk patients from other medical conditions.

b) no

Comparability

1) Comparability of cohorts on the basis of the design or analysis

a) study controls for any additional various sociodemographic and lifestyle variables*

b) study controls for any additional various sociodemographic and lifestyle variables*

psychosocial covariates found to be related to pet ownership (age, marital status, years of education, self-reported regular exercise) and variables found to predict survival in a previous analysis; post-ACS onset depression, diabetes on admission, cerebrovascular accident (CVA) or

transient ischemic attack (TIA) on admission, LVEF (left ventricular ejection fraction less than 35%) and having a Coronary Artery Bypass Graft (CABG) during baseline admission.

Outcome

1) Assessment of outcome

a) independent blind assessment *

b) record linkage *

Names of all untraced individuals were submitted to the New South Wales (NSW) Registry of Births, Deaths and Marriages and The National Death Index, allowing mortality status to be established.

c) self report

d) no description

2) Was follow-up long enough for outcomes to occur

a) yes (select an adequate follow up period for outcome of interest) 1 year following hospitalization

b) no

3) Adequacy of follow up of cohorts

a) complete follow up - all subjects accounted for *

b) subjects lost to follow up unlikely to introduce bias - small number lost - > ____ % (select an adequate %) follow up, or description provided of those lost) *

c) follow up rate < ____% (select an adequate %) and no description of those lost

d) no statement

NEWCASTLE - OTTAWA QUALITY ASSESSMENT SCALE

COHORT STUDIES Guillum 2010

Note: A study can be awarded a maximum of one star for each numbered item within the Selection and Outcome categories. A maximum of two stars can be given for Comparability

Selection

1) Representativeness of the exposed cohort

a) truly representative of the average __population_ in the community *

The Third National Health and Nutrition Examination Survey (NHANES III) was conducted in 1988–1994 on a nationwide multi-stage probability sample of 39,695 persons from the civilian, non-institutionalized population aged 2 months and over of the United States.

b) somewhat representative of the average _in the community *

c) selected group of users eg nurses, volunteers

d) no description of the derivation of the cohort

2) Selection of the non exposed cohort

a) drawn from the same community as the exposed cohort *

b) drawn from a different source

c) no description of the derivation of the non-exposed cohort

3) Ascertainment of exposure

a) secure record (eg surgical records) *

b) structured interview *During a home interview, interviewers asked questions concerning animals living in the household

c) written self-report

d) no description

4) Demonstration that outcome of interest was not present at start of study

a) yes *

b) no, the secondary survey did not mention the exclusion criteria, if other high risk of sudden death or mortality exist in the start

Comparability

1) Comparability of cohorts on the basis of the design or analysis

a) study controls for _the most important factor with pet ownership *

model 1: age, sex, race, living with pet

b) study controls for any additional various sociodemographic and lifestyle variables*

model 1: age, sex, race, living with pet

model 2: socioeconomic health status and activity

model 3: healthy behaviors and other risk factors

Outcome

1) Assessment of outcome

a) independent blind assessment *

b) record linkage *

To obtain the mortality outcome variables, a mortality linkage was done based upon the results from a probabilistic match of NHANES III participants with the National Death Index.

c) self-report

d) no description

2) Was follow-up long enough for outcomes to occur

a) yes (select an adequate follow up period for outcome of interest) 8.5 years

3) Adequacy of follow up of cohorts

d) no statement

NEWCASTLE - OTTAWA QUALITY ASSESSMENT SCALE

COHORT STUDIES Qureshi 2009

Note: A study can be awarded a maximum of one star for each numbered item within the Selection and Outcome categories. A maximum of two stars can be given for Comparability

Selection

1) Representativeness of the exposed cohort

a) truly representative of the average __population_ in the community *

National Health and Nutrition Examination Study (NHANES II) Follow-up Study5

b) somewhat representative of the average _in the community *

c) selected group of users eg nurses, volunteers

d) no description of the derivation of the cohort

2) Selection of the non exposed cohort

a) drawn from the same community as the exposed cohort *

b) drawn from a different source

c) no description of the derivation of the non-exposed cohort

3) Ascertainment of exposure

a) secure record (eg surgical records) *

b) structured interview *

At baseline evaluation of National Health and Nutrition Examination Study (NHANES II) Follow-up Study5, any participant who reported any kind of allergy was queried further whether they owned or currently own a cat or a dog.

c) written self-report

d) no description

4) Demonstration that outcome of interest was not present at start of study

a) yes *

Events of ischemic stroke, intracerebral hemorrhage (ICH), and myocardial infarction (MI) during follow up were determined by ICD.

b) no, the secondary survey did not mention the exclusion criteria, if other high risk of sudden death or mortality exist in the start

Comparability

1) Comparability of cohorts on the basis of the design or analysis

a) study controls for _the most important factor variables with pet ownership *

b) study controls for any additional various sociodemographic and lifestyle variables* adjusted for age, gender, ethnicity/race, systolic blood pressure, cigarette smoking, diabetes mellitus, serum cholesterol, BMI

Outcome

1) Assessment of outcome

a) independent blind assessment *

b) record linkage * (ICD-9 CM) diagnosis codes:

c) self report

d) no description

2) Was follow-up long enough for outcomes to occur

a) yes (select an adequate follow up period for outcome of interest) 13.4±3.6 years.

b) no

3) Adequacy of follow up of cohorts

a) complete follow up - all subjects accounted for *

b) subjects lost to follow up unlikely to introduce bias - small number lost - > ____ % (select an adequate %) follow up, or description

c) follow up rate < ____% (select an adequate %) and no description of those lost

d) no statement

NEWCASTLE - OTTAWA QUALITY ASSESSMENT SCALE

COHORT STUDIES Friedman 1995

Note: A study can be awarded a maximum of one star for each numbered item within the Selection and Outcome categories. A maximum of two stars can be given for Comparability

Selection

1) Representativeness of the exposed cohort

a) truly representative of the average __population_ in the community *

b) somewhat representative of the average cardiac arrhythmia patient_in the community *

all patients entered CAST or CASTII are potential eligible for the study, pharmacological tests of the arrhythmia suppression and mortality hypothesis.

c) selected group of users eg nurses, volunteers

d) no description of the derivation of the cohort

2) Selection of the non exposed cohort

a) drawn from the same community as the exposed cohort *

b) drawn from a different source

c) no description of the derivation of the non-exposed cohort

3) Ascertainment of exposure

a) secure record (eg surgical records) *

b) structured interview *

c) written self-report questionnaire

d) no description

4) Demonstration that outcome of interest was not present at start of study

a) yes *

b) no, the secondary survey did not mention the exclusion criteria, if other high risk of sudden death or mortality exist in the start

Comparability

1) Comparability of cohorts on the basis of the design or analysis

a) study controls for study controls for _the most important factor variables with pet ownership *

b) study controls for any additional physiologic and other psychosocial variables*

In the logistic regression analysis, when the effects of physiologic and other psychosocial variables were controlled, pet owners tended to be more likely to survive 1 year than nonowners. (The description was not specifically mention what factors are adjusted)

Outcome

1) Assessment of outcome

a) independent blind assessment *

b) record linkage *

Mortality data were obtained by CAST clinical site personnel from physicians, family members, and medical records.

c) self report

d) no description

2) Was follow-up long enough for outcomes to occur

a) yes (select an adequate follow up period for outcome of interest) 1 year after baseline

b) no

3) Adequacy of follow up of cohorts

b) subjects lost to follow up unlikely to introduce bias - small number lost - > _55/424___ % *

A total of 424 subjects were recruited into the study. One-year survival status was obtained from 369 participants (87%). Of the 55 participants lost to follow-up, 54 (98%) had been followed for ~1 year when

CAST II ended prematurely.

NEWCASTLE - OTTAWA QUALITY ASSESSMENT SCALE

COHORT STUDIES Friedman 1980

Note: A study can be awarded a maximum of one star for each numbered item within the Selection and Outcome categories. A maximum of two stars can be given for Comparability

Selection

1) Representativeness of the exposed cohort

a) truly representative of the average _ _ in the community *

b) somewhat representative of the average white MI or angina patients in the community*.

c) selected group of users eg nurses, volunteers

All white patients with a diagnosis of MI or AP on admission to the coronary care unit, medical intensive care unit, or coronary care stepdown unit of a large university hospital, between August 1975 and March 1977, were invited to participate in this study

d) no description of the derivation of the cohort

2) Selection of the non exposed cohort

a) drawn from the same community as the exposed cohort *

b) drawn from a different source

c) no description of the derivation of the non-exposed cohort

3) Ascertainment of exposure

a) secure record (eg surgical records) *

b) structured interview *

c) written self-report questionnaires

d) no description

4) Demonstration that outcome of interest was not present at start of study

a) yes *

b) no, the survey did not mention the exclusion criteria, if other high risk of sudden death or mortality exist in the start

Comparability

1) Comparability of cohorts on the basis of the design or analysis

a) study controls for the most important variables *

b) study controls for any additional physiologic and other psychosocial variables*

not controlled

Outcome

1) Assessment of outcome

a) independent blind assessment *

b) record linkage *

Each death was confirmed by hospital records or by interviews with the next of kin, and the cause of death was noted.

c) self-report

d) no description

2) Was follow-up long enough for outcomes to occur

a) yes (select an adequate follow up period for outcome of interest) 1 year after baseline

b) no

3) Adequacy of follow up of cohorts

a) complete follow up - all subjects accounted for *

b) subjects lost to follow up unlikely to introduce bias - small number lost - > _2/92_ % follow up, or description provided of those lost) * After 1 year, we contacted all the surviving patients; two patients could not be located.

c) follow up rate < ____% (select an adequate %) and no description of those lost

d) no statement

NEWCASTLE - OTTAWA QUALITY ASSESSMENT SCALE FOR CROSS-SECTIONAL STUDIES Xie 2017

Selection:

1.Representativeness of the sample:

a. Truly representative of the average in the target population. * (all subjects or random sampling)

All patients suffered from typical or atypical chest pain or abnormal ST-T changes, and they were admitted to this hospital for coronary arteriography.

b. Somewhat representative of the average in the target group. * (non-random sampling)

c. Selected group of users/convenience sample.

d. No description of the derivation of the included subjects.

2. Sample size:

a. Justified and satisfactory (including sample size calculation). *

Finally, 376 males and 185 females were enrolled in this study.

b. Not justified.

c. No information provided

3. Non-respondents:

a. Proportion of target sample recruited attains pre-specified target or basic summary of non-respondent characteristics in sampling frame recorded. *

Two participants were excluded because their pets were not dogs or cats. Moreover, 12 patients were also excluded as they refused to participate in this survey.

b. Unsatisfactory recruitment rate, no summary data on non-respondents.

c. No information provided

4. Ascertainment of the exposure (risk factor):

a. Vaccine records/vaccine registry/clinic registers/hospital records only. **

b. Parental or personal recall and vaccine/hospital records. *

pet ownership information was obtained with the help of a quantitative questionnaire

c. Parental/personal recall only.

Comparability: (Maximum 2 stars)

1. Comparability of subjects in different outcome groups on the basis of design or analysis. Confounding factors controlled.

a. Data/ results adjusted for relevant predictors/risk factors/confounders e.g. age, sex, time since vaccination, etc. **

Adjustment for age, gender, body mass index, hypertension, diabetes mellitus, hyperlipidemia, smoking status, alcohol use, physical activity, and education in the analysis

b. Data/results not adjusted for all relevant confounders/risk factors/information not provided.

Outcome:

1. Assessment of outcome:

a. Independent blind assessment using objective validated laboratory methods. **

All patients underwent coronary arteriography by 2 experienced angiographers using the standard Judkins technique, a selective coronary arteriography technique that was performed via femoral artery puncture. All the assessments were in accordance with the American Heart Association method. The evaluators were blind to pet ownership information. CAD was defined if there was stenosis ≥50% in any of major coronary
arteries (including left main, left anterior descending, left circumflex, and right coronary artery).

b. Unblinded assessment using objective validated laboratory methods. **

c. Used non-standard or non-validated laboratory methods with gold standard. *

d. No description/non-standard laboratory methods used.

2. Statistical test:

a. Statistical test used to analyse the data clearly described, appropriate and measures of association presented including confidence intervals and probability level (p value). *

Characteristics were compared between groups using chi-square tests for categorical variables. Independent-samples t test was used for continuous variables. Univariate and multiple logistic regression
analyses were used to access and exclude various factors influencing the interference.

All P values were 2-sided and should be less than 0.05. Bonferroni correction was applied in multiple comparisons when using chisquare tests for categorical variables. To be exact, it was statistically significant only if the P value for multiple comparisons was lower than 0.017 in smoking and drinking status or 0.005 in education level.

b. Statistical test not appropriate, not described or incomplete.

Cross-sectional Studies:

Very Good Studies: 9-10 points

Good Studies: 7-8 points

Satisfactory Studies: 5-6 points

Unsatisfactory Studies: 0 to 4 points
