# Supplementary material for: A modest protective association between pet ownership and cardiovascular diseases: A systematic review and meta-analysis
Source: PLoS One. 2019 May 3;14(5):e0216231. doi: 10.1371/journal.pone.0216231 (PMC6499429; doi:10.1371/journal.pone.0216231)

S1 Fig A. Meta-regression between pet type and adjusted all-cause mortality.

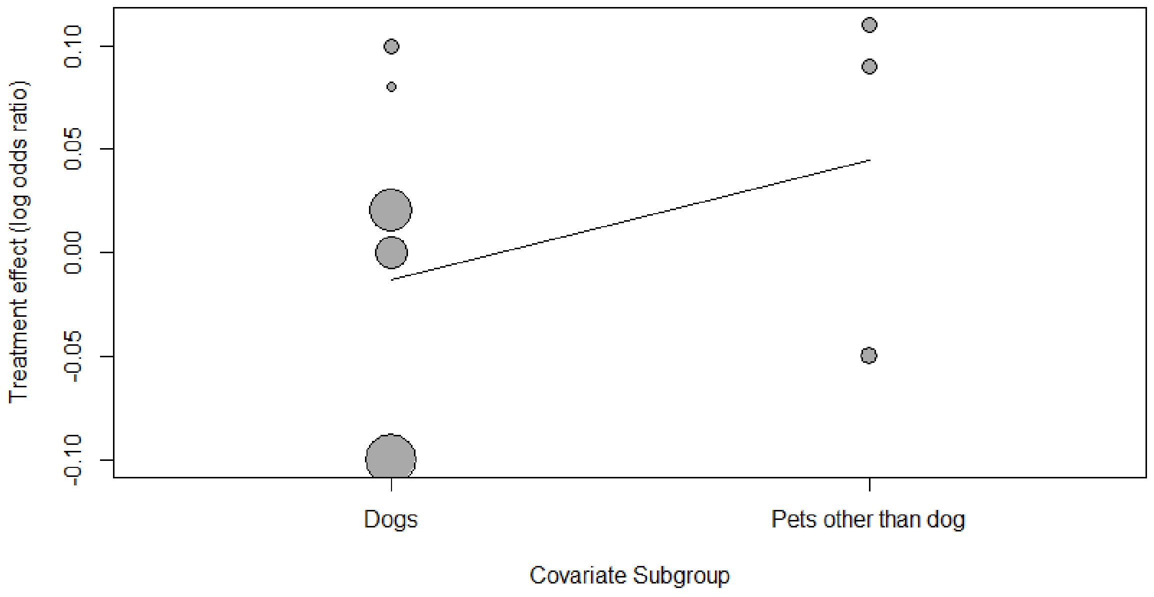

S1 Fig B. Meta-regression between pet type and adjusted CV mortality.

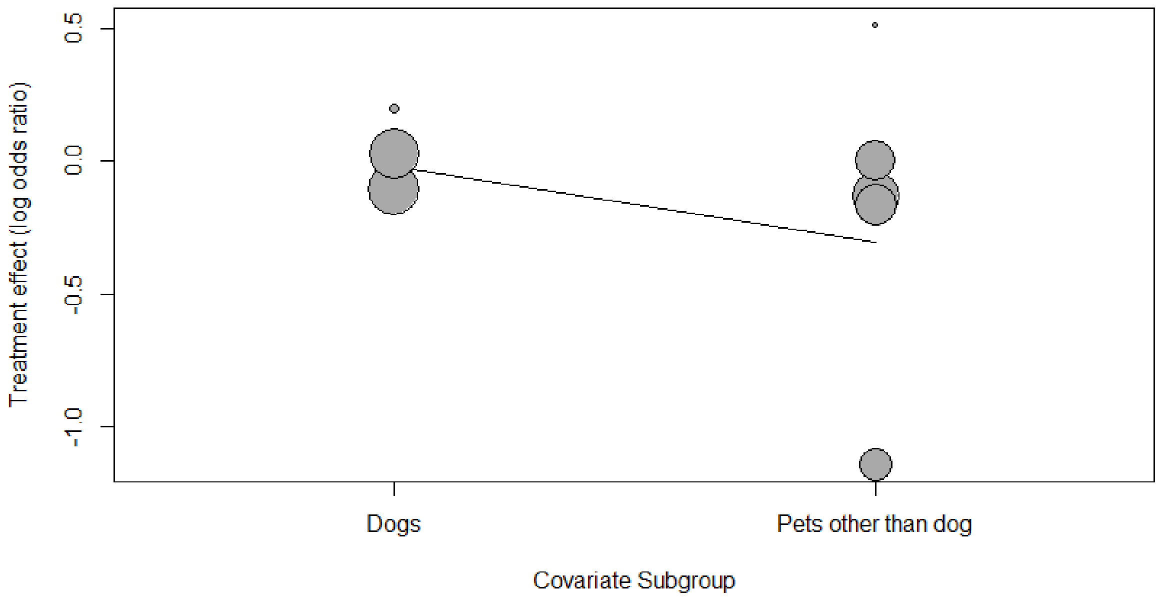

S1 Fig C. Meta-regression between pet type and adjusted CVD.

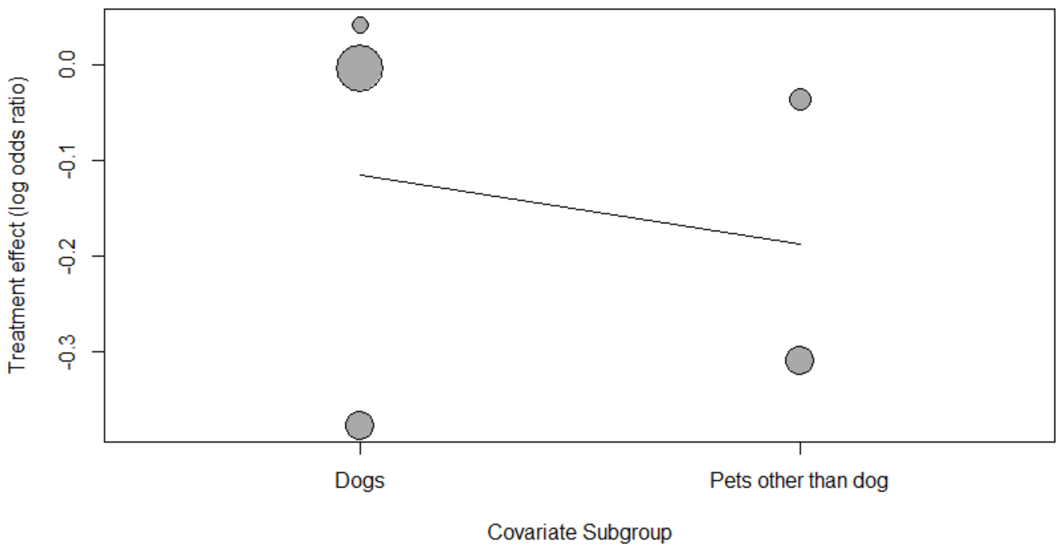

S1 Fig D. Meta-regression between pet type and adjusted MI.

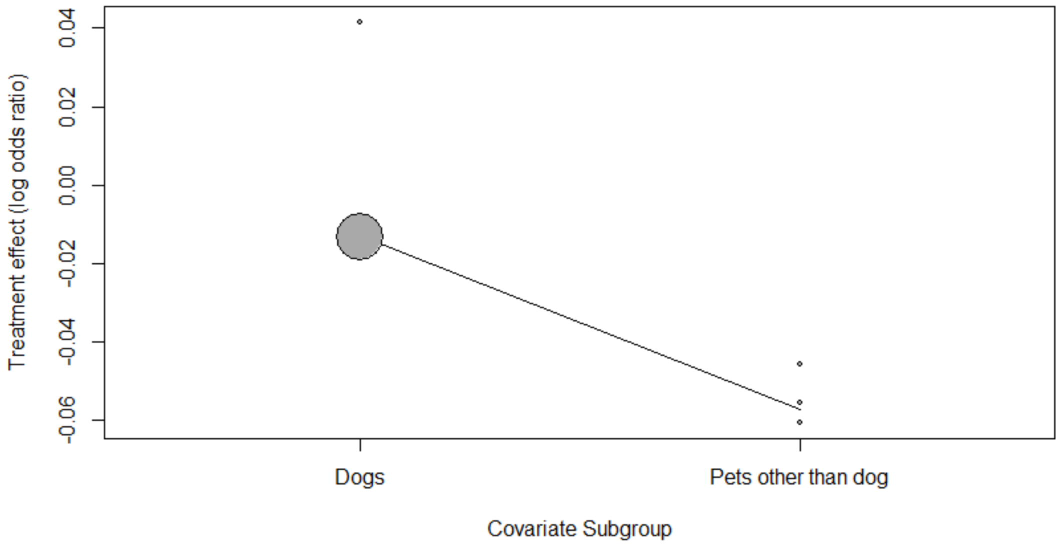

S1 Fig E. Meta-regression between pet type and adjusted stroke.

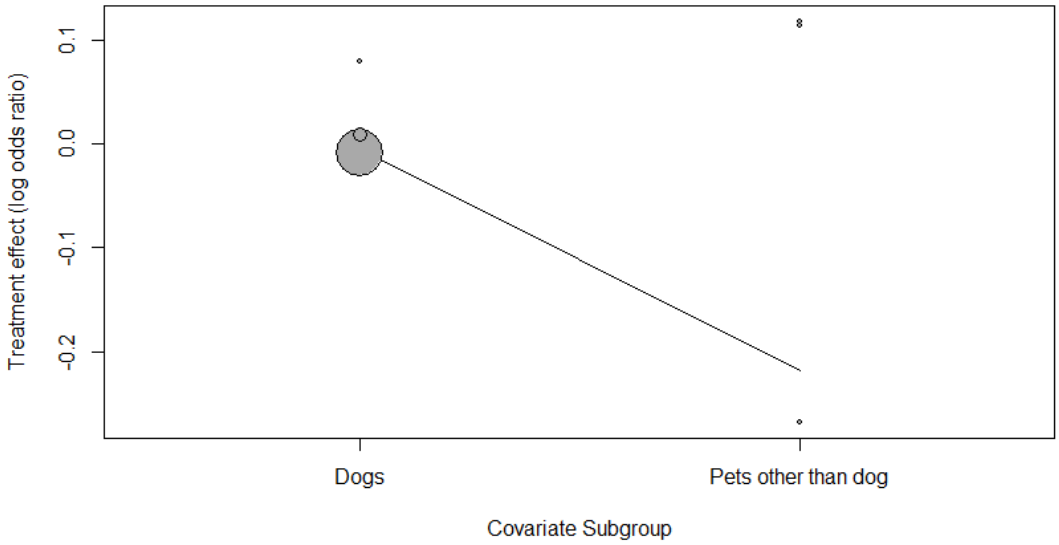

Supplement: S1 Fig — (PDF) [file pone.0216231.s006.pdf]
