# Supplementary material for: A modest protective association between pet ownership and cardiovascular diseases: A systematic review and meta-analysis
Source: PLoS One. 2019 May 3;14(5):e0216231. doi: 10.1371/journal.pone.0216231 (PMC6499429; doi:10.1371/journal.pone.0216231)
Supplement: S2 Fig — (PDF) [file pone.0216231.s007.pdf]

S2 Fig A. The funnel plot of adjusted all-cause mortality.

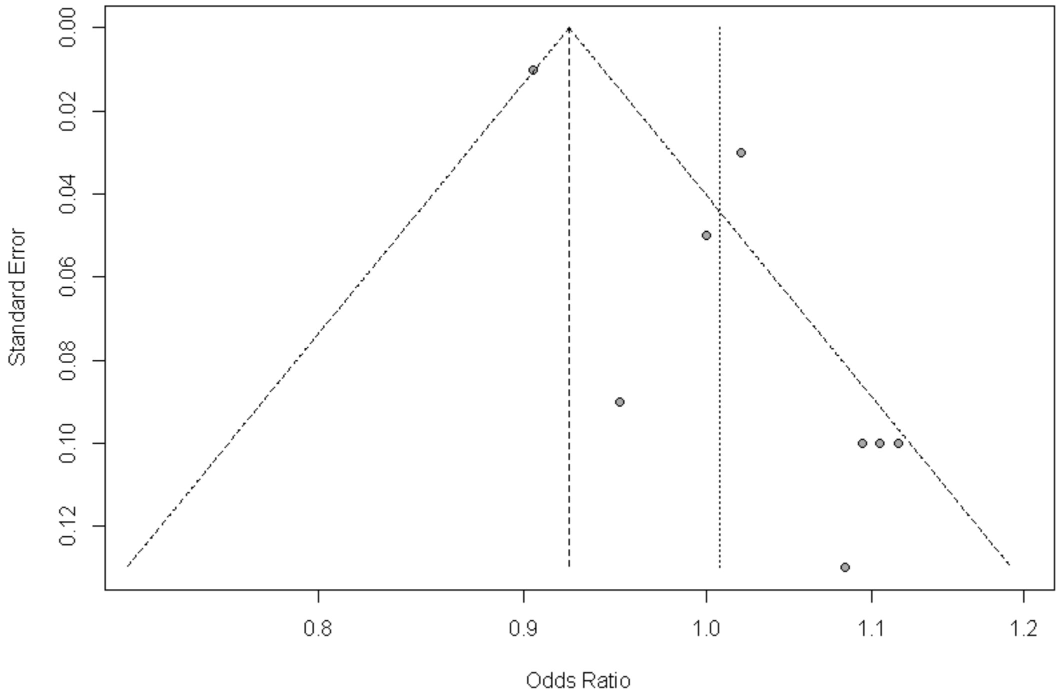

Egger's test

- t = 3.8251, df = 6, p-value = 0.008708
- alternative hypothesis: asymmetry in funnel plot
- sample estimates:
- bias se.bias slope
- 2.1170352 0.5534539 -0.1148222

S2 Fig B. The funnel plot of adjusted CV mortality.

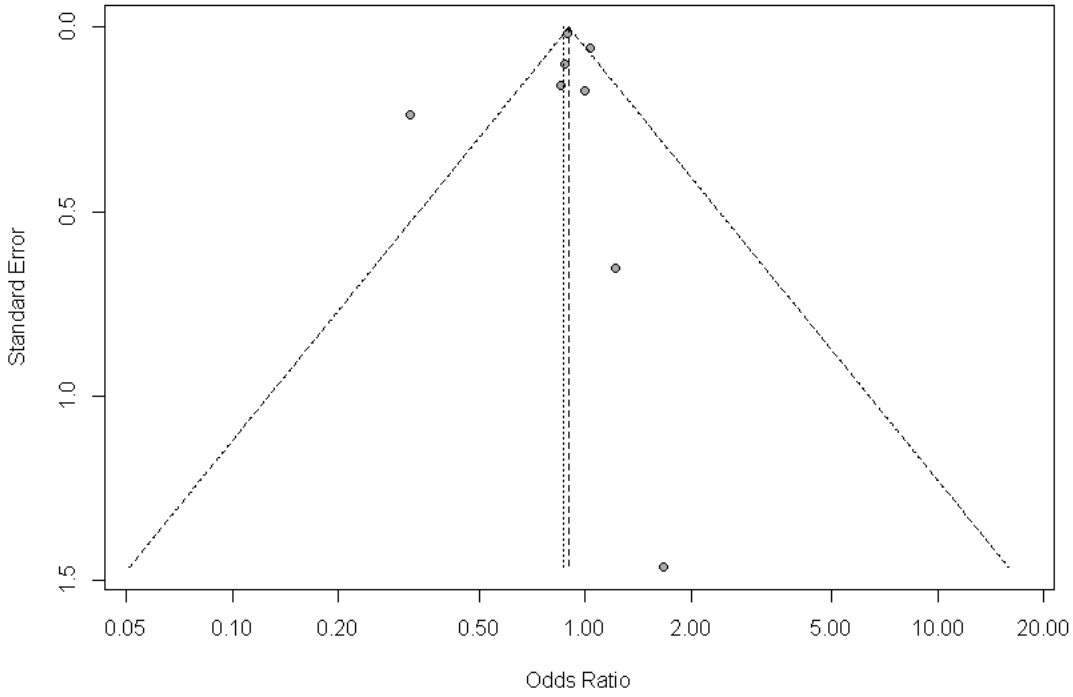

Egger's test

- t = -0.36375, df = 6, p-value = 0.7285
- alternative hypothesis: asymmetry in funnel plot
- sample estimates:
- bias se.bias slope
- -0.32786538 0.90135415 -0.09213315

S2 Fig C. The funnel plot of adjusted CVD.

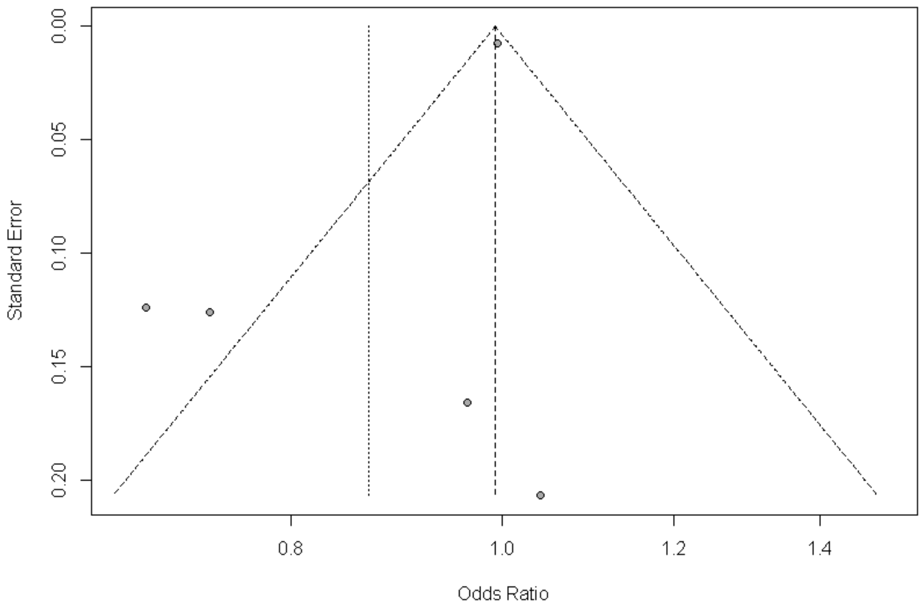

Egger's test

- t = -1.6373, df = 3, p-value = 0.2001
- alternative hypothesis: asymmetry in funnel plot
- sample estimates:
- bias se.bias slope
- -1.403209418 0.857003894 0.005918752

S2 Fig D. The funnel plot of adjusted MI.

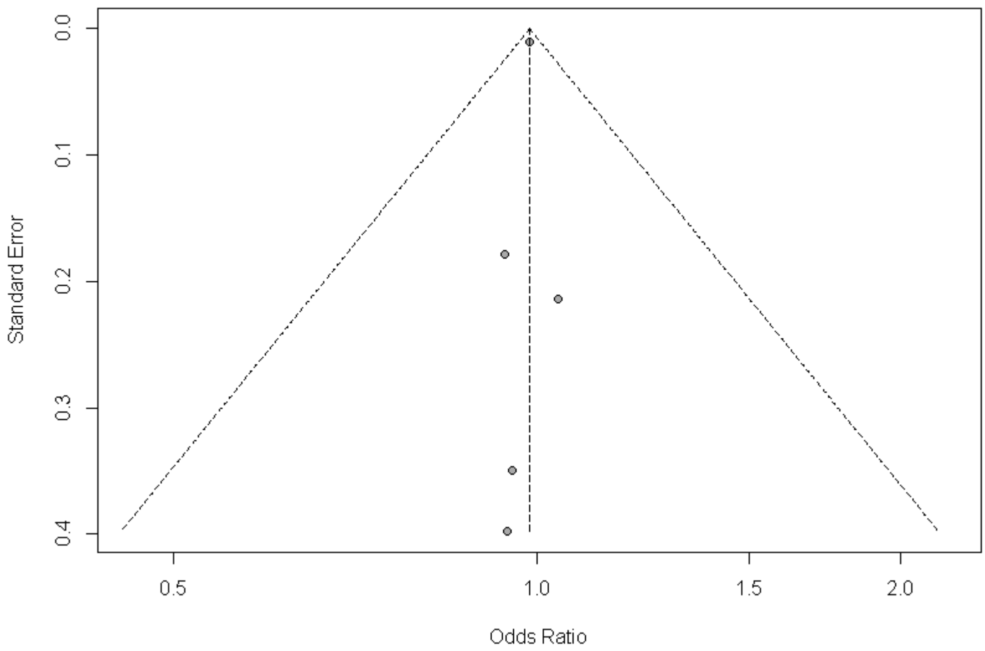

Egger's test

- t = -0.47527, df = 3, p-value = 0.6671
- alternative hypothesis: asymmetry in funnel plot
- sample estimates:
- bias se.bias slope
- -0.05439848 0.11445908 -0.01267446

S2 Fig E. The funnel plot of adjusted CVA.

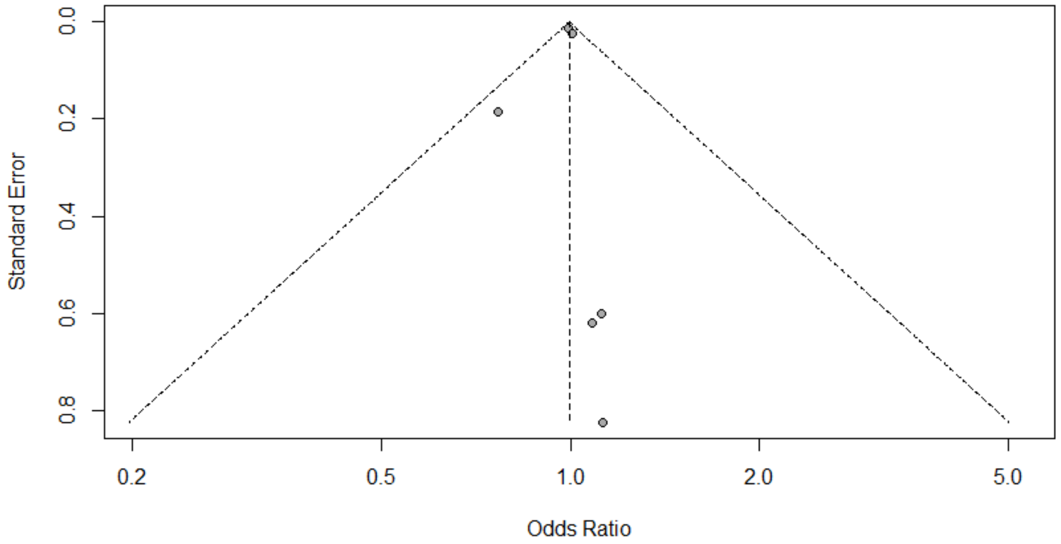

.

Egger's test

- t = -0.36859, df = 4, p-value = 0.7311
- alternative hypothesis: asymmetry in funnel plot
- sample estimates:
- bias se.bias slope
- -0.146181828 0.396602518 -0.003139978
