# Supplementary material for: A modest protective association between pet ownership and cardiovascular diseases: A systematic review and meta-analysis
Source: PLoS One. 2019 May 3;14(5):e0216231. doi: 10.1371/journal.pone.0216231 (PMC6499429; doi:10.1371/journal.pone.0216231)

**S3 Fig A. Sensitivity test of adjusted cardiovascular mortality through omitting Parker 2010 (dog).**

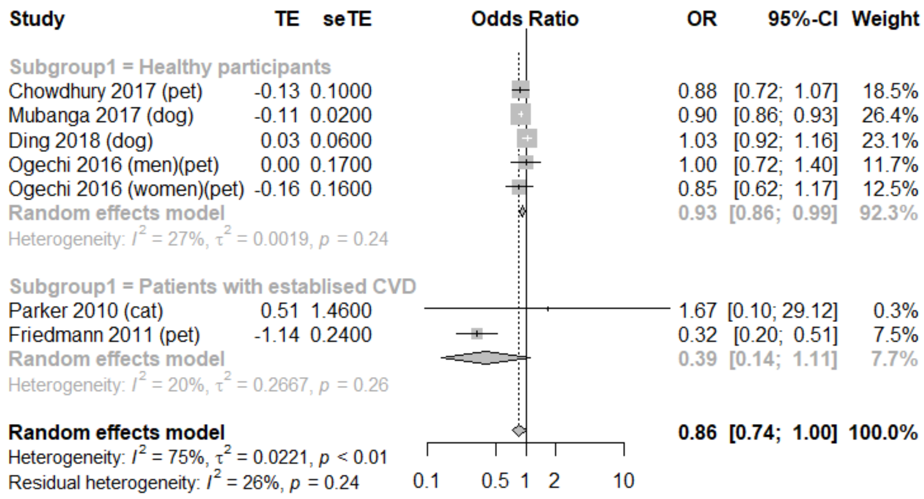


**S3 Fig B. Sensitivity test of adjusted cardiovascular mortality through omitting Parker 2010 (cat).**

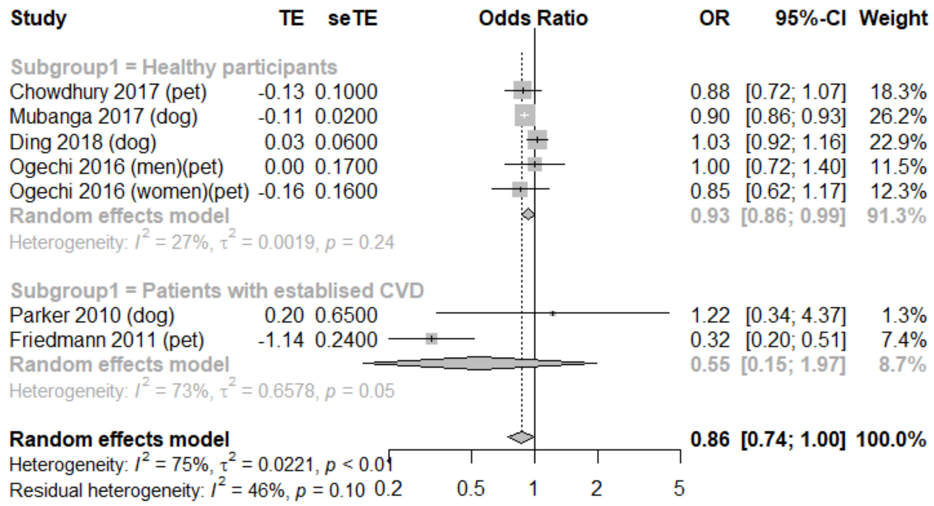

## S3 Fig C. Sensitivity test of adjusted cardiovascular mortality through omitting Friedmann 2011 (pet).

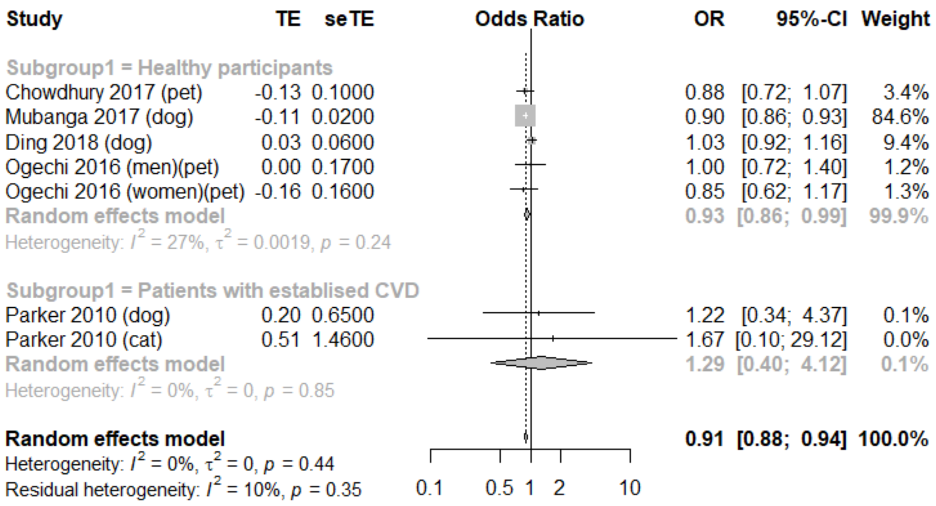


## S3 Fig D. Sensitivity test of adjusted cardiovascular mortality through omitting Chowdhury 2017 (pet)

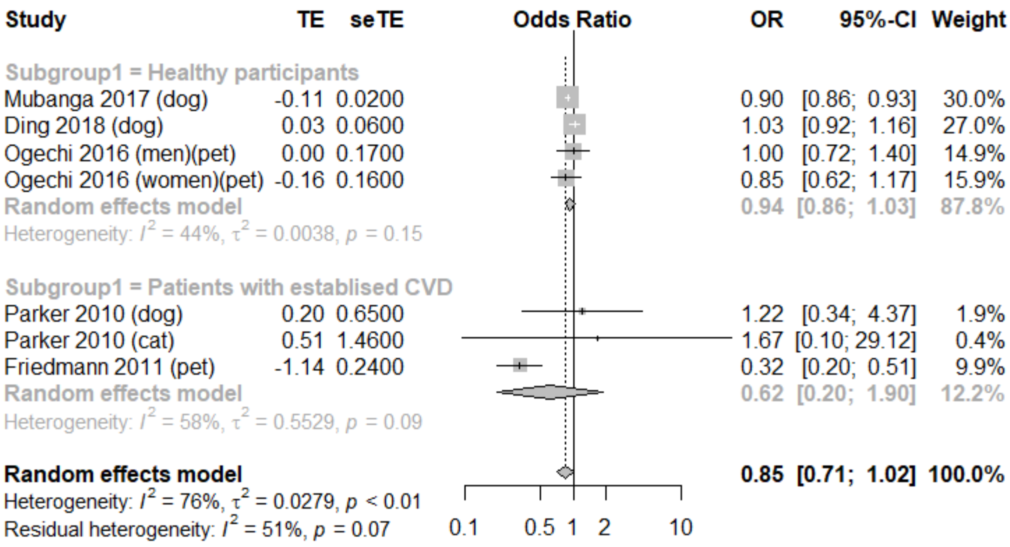

**S3 Fig E. Sensitivity test of adjusted cardiovascular mortality through omitting Mubanga 2017 (dog)**

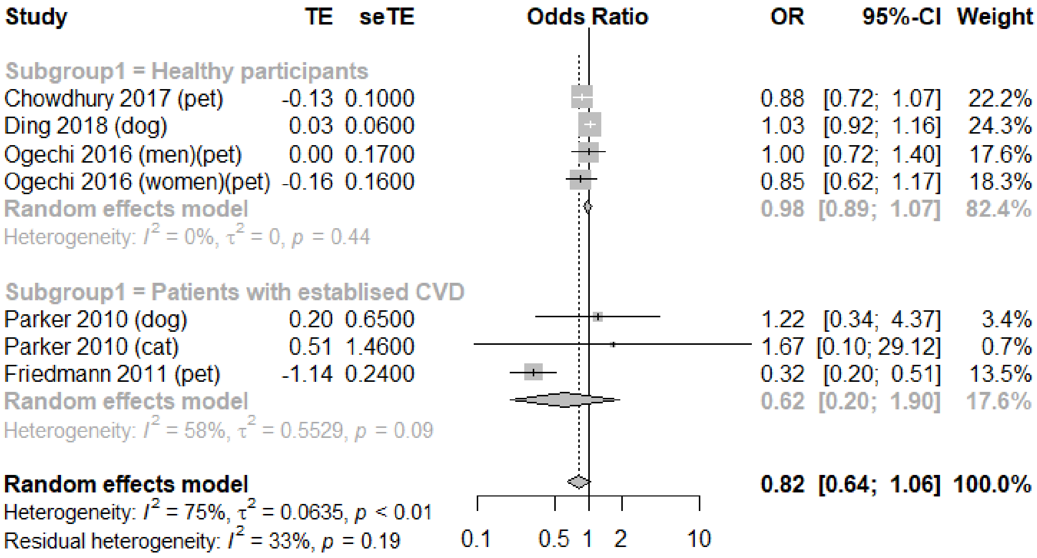


**S3 Fig F. Sensitivity test of adjusted cardiovascular mortality through omitting Ding 2018 (dog)**

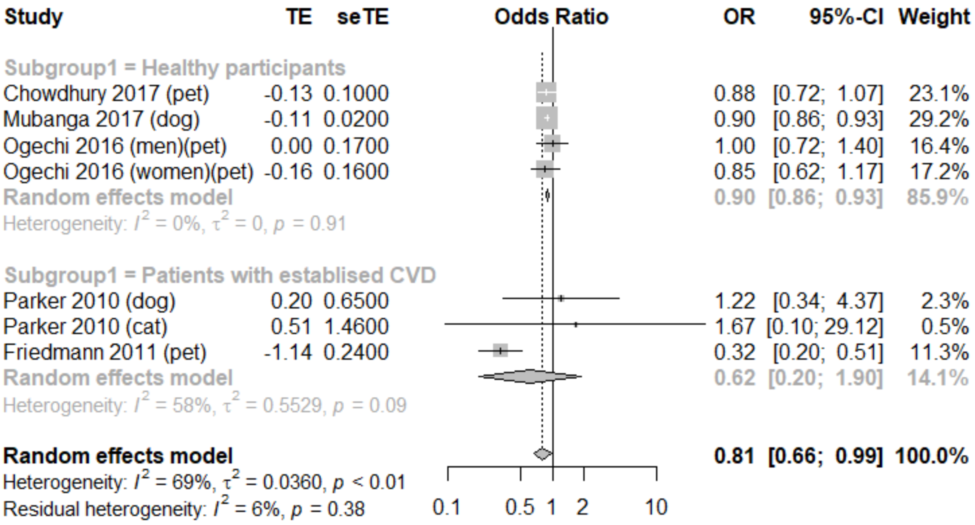

**S3 Fig G. Sensitivity test of adjusted cardiovascular mortality through omitting Ogechi 2016 (men)(pet)**

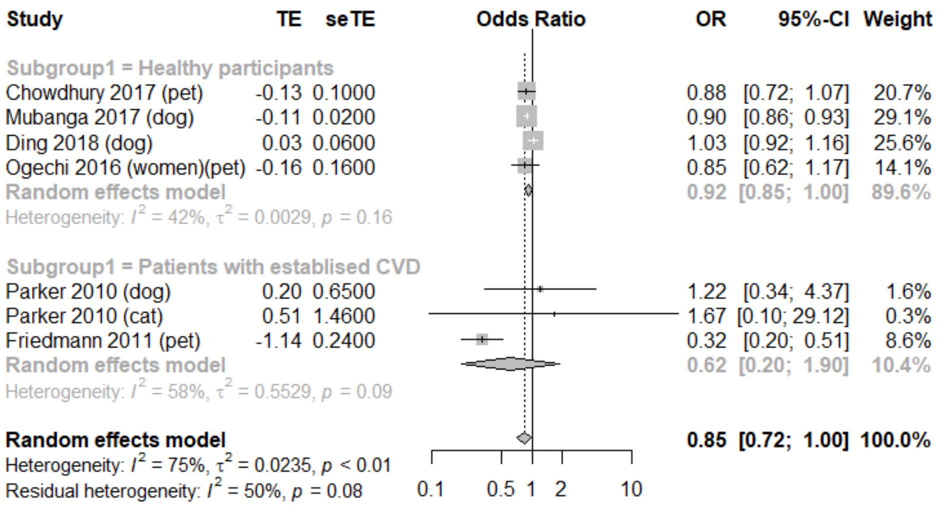


**S3 Fig H. Sensitivity test of adjusted cardiovascular mortality through omitting Ogechi 2016 (women)(pet)**

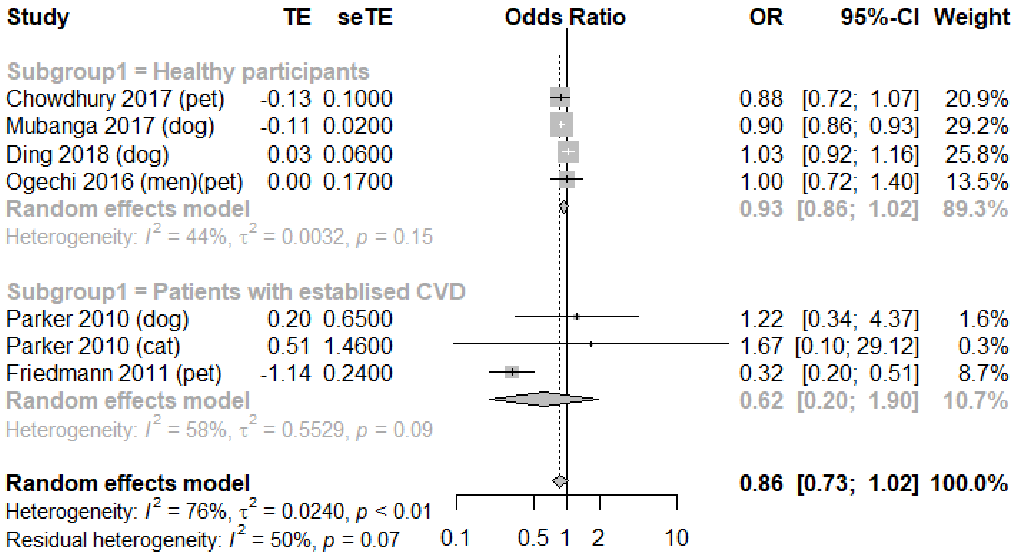

## S3 Fig I. Sensitivity test of risk of cardiovascular disease through omitting Qureshi 2009 (cat)

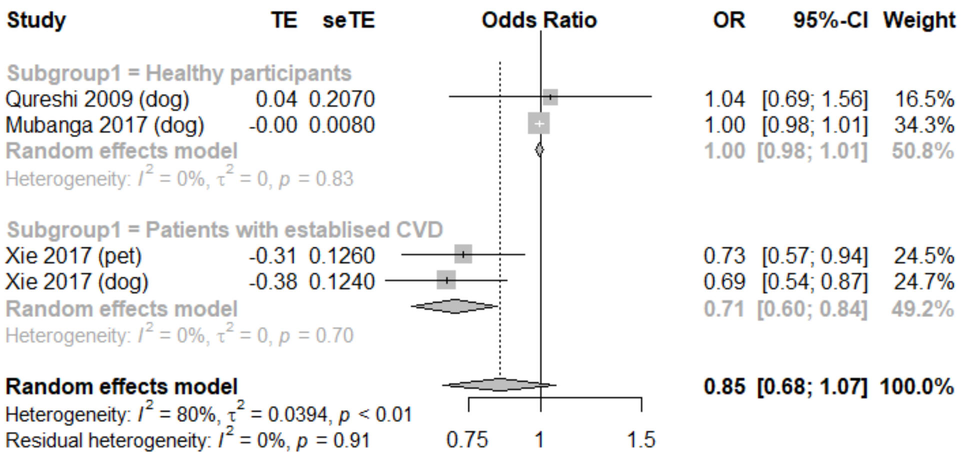


## S3 Fig J. Sensitivity test of risk of cardiovascular disease through omitting Qureshi 2009 (dog)

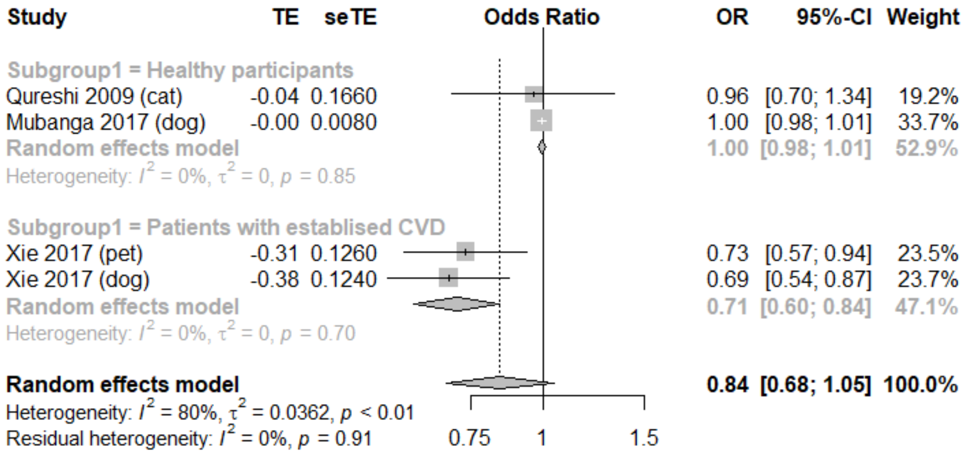

**S3 Fig K. Sensitivity test of risk of cardiovascular disease through omitting Mubanga 2017 (dog)**

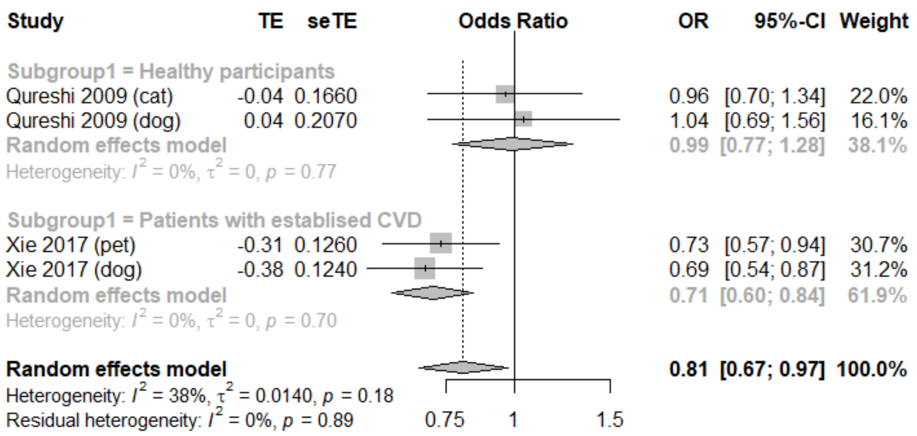


**S3 Fig L. Sensitivity test of risk of cardiovascular disease through omitting Xie 2017 (pet)**

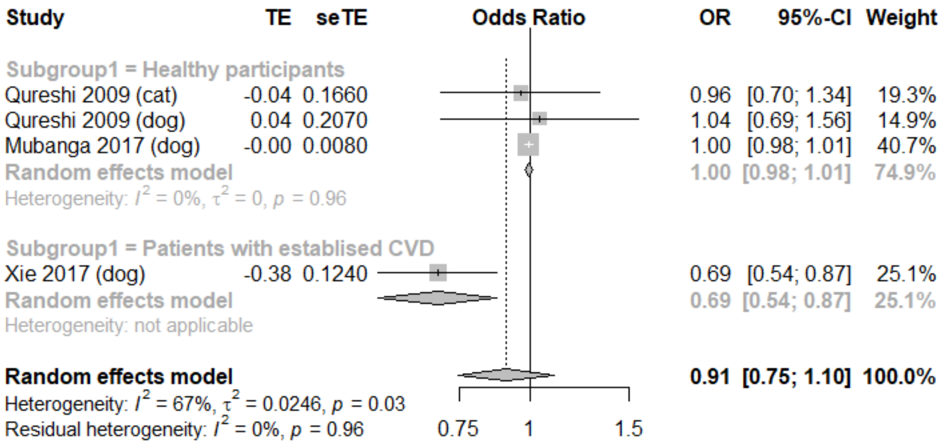

## S3 Fig M. Sensitivity test of risk of cardiovascular disease through omitting Xie 2017 (dog)

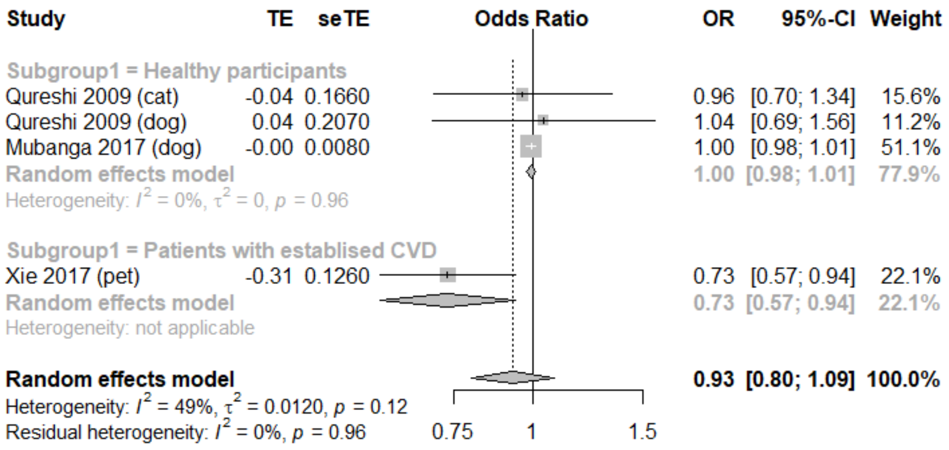

Supplement: S3 Fig — (PDF) [file pone.0216231.s008.pdf]
