# Supplementary material for: A modest protective association between pet ownership and cardiovascular diseases: A systematic review and meta-analysis
Source: PLoS One. 2019 May 3;14(5):e0216231. doi: 10.1371/journal.pone.0216231 (PMC6499429; doi:10.1371/journal.pone.0216231)
Supplement: S1 Dataset — (PDF) [file pone.0216231.s009.pdf]

R code

```
install.packages("meta”)
library(metagen)
> alldeath<-read.delim("clipboard")
> alldeath
> meta1<-metagen(TE, seTE, studlab = Article,sm="OR",data=alldeath)
> forest(meta1)
> meta11<-metagen(TE, seTE, studlab = Article,sm="OR",data=alldeath, byvar =
Subgroup)
> meta12<-metagen(TE, seTE, studlab = Article,sm="OR",data=alldeath, byvar =
Subgroup1)
forest(meta11,comb.fixed=F)
forest(meta12 ,comb.fixed=F)
library(metafor)
meta1r<-metareg(meta1,~Subgroup, method.tau="REML", hakn = TRUE)
bubble(meta1r)
metabias(meta1, k.min=8 ,method.bias="linreg", plotit=T)

CVdeath<-read.delim("clipboard")
> CVdeath
> meta2<-metagen(TE, seTE, studlab = Article,sm="OR",data=CVdeath)
>    meta21<-metagen(TE, seTE, studlab = Article,sm="OR",data=CVdeath, byvar =
Subgroup)
> meta22<-metagen(TE, seTE, studlab = Article,sm="OR",data=CVdeath, byvar =
Subgroup1)
forest(meta21,comb.fixed=F)
forest(meta22 ,comb.fixed=F)
meta2r<-metareg(meta2,~Subgroup, method.tau="REML", hakn = TRUE)
bubble(meta2r)
funnel(meta2, comb.random = F)
metabias(meta2, k.min=8 ,method.bias="linreg", plotit=T)

> CVD<-read.delim("clipboard")
> CVD
meta3<-metagen(TE, seTE, studlab = Article,sm="OR",data=CVD)
meta31<-metagen(TE, seTE, studlab = Article,sm="OR",data=CVD, byvar = Subgroup)
meta32<-metagen(TE, seTE, studlab = Article,sm="OR",data=CVD, byvar =
Subgroup1)
```

```
forest(meta31,comb.fixed=F)
forest(meta32 ,comb.fixed=F)
meta3r<-metareg(meta3,~Subgroup, method.tau="REML", hakn = TRUE)
bubble(meta3r)
funnel(meta3, comb.random = F)
metabias(meta3, k.min=5 ,method.bias="linreg", plotit=T)

MI<-read.delim("clipboard")
MI
meta4<-metagen(TE, seTE, studlab = Article,sm="OR",data=MI)
meta41<-metagen(TE, seTE, studlab = Article,sm="OR",data=MI, byvar = Subgroup)
meta42<-metagen(TE, seTE, studlab = Article,sm="OR",data=MI, byvar = Subgroup1)
forest(meta4,comb.fixed=F)
forest(meta42 ,comb.fixed=F)
meta4r<-metareg(meta4,~Subgroup, method.tau="REML", hakn = TRUE)
bubble(meta4r)
funnel(meta4, comb.random = F)
metabias(meta4, k.min=5 ,method.bias="linreg", plotit=T)

CVA<-read.delim("clipboard")
> CVA
meta5<-metagen(TE, seTE, studlab = Article,sm="OR",data=CVA)
meta51<-metagen(TE, seTE, studlab = Article,sm="OR",data=CVA, byvar = Subgroup)
meta52<-metagen(TE, seTE, studlab = Article,sm="OR",data=CVA, byvar =
Subgroup1)
forest(meta5,comb.fixed=F)
forest(meta52 ,comb.fixed=F)
meta5r<-metareg(meta5,~Subgroup, method.tau="REML", hakn = TRUE)
bubble(meta5r)
funnel(meta5, comb.random = F)
metabias(meta5, k.min=6 ,method.bias="linreg", plotit=T)

install.packages("meta")
library(meta)

s21<-read.delim("clipboard")
s21
metas21<-metagen(TE, seTE, studlab=Article, sm="OR", data=s21,byvar=Subgroup1)
```

```
forest(metas21,comb.fixed=F)

s22<-read.delim("clipboard")
s22
metas22<-metagen(TE, seTE, studlab=Article, sm="OR", data=s22,byvar=Subgroup1)
forest(metas22,comb.fixed=F)

s23<-read.delim("clipboard")
s23
metas23<-metagen(TE, seTE, studlab=Article, sm="OR", data=s23,byvar=Subgroup1)
forest(metas23,comb.fixed=F)

s24<-read.delim("clipboard")
s24
metas24<-metagen(TE, seTE, studlab=Article, sm="OR", data=s24,byvar=Subgroup1)
forest(metas24,comb.fixed=F)

s25<-read.delim("clipboard")
s25
metas25<-metagen(TE, seTE, studlab=Article, sm="OR", data=s25,byvar=Subgroup1)
forest(metas25,comb.fixed=F)

s26<-read.delim("clipboard")
s26
metas26<-metagen(TE, seTE, studlab=Article, sm="OR", data=s26,byvar=Subgroup1)
forest(metas26,comb.fixed=F)

s27<-read.delim("clipboard")
s27
metas27<-metagen(TE, seTE, studlab=Article, sm="OR", data=s27,byvar=Subgroup1)
forest(metas27,comb.fixed=F)

s28<-read.delim("clipboard")
s28
metas28<-metagen(TE, seTE, studlab=Article, sm="OR", data=s28,byvar=Subgroup1)
forest(metas28,comb.fixed=F)

s31<-read.delim("clipboard")
```

```
s31
metas31<-metagen(TE, seTE, studlab=Article, sm="OR", data=s31,byvar=Subgroup1)
forest(metas31,comb.fixed=F)

s32<-read.delim("clipboard")
s32
metas32<-metagen(TE, seTE, studlab=Article, sm="OR", data=s32,byvar=Subgroup1)
forest(metas32,comb.fixed=F)

s33<-read.delim("clipboard")
s33
metas33<-metagen(TE, seTE, studlab=Article, sm="OR", data=s33,byvar=Subgroup1)
forest(metas33,comb.fixed=F)

s34<-read.delim("clipboard")
s34
metas34<-metagen(TE, seTE, studlab=Article, sm="OR", data=s34,byvar=Subgroup1)
forest(metas34,comb.fixed=F)

s35<-read.delim("clipboard")
s35
metas35<-metagen(TE, seTE, studlab=Article, sm="OR", data=s35,byvar=Subgroup1)
forest(metas35,comb.fixed=F)
```
